# Effect of Insulin Resistance on Monounsaturated Fatty Acid Levels: A Multi-cohort Non-targeted Metabolomics and Mendelian Randomization Study

**DOI:** 10.1371/journal.pgen.1006379

**Published:** 2016-10-21

**Authors:** Christoph Nowak, Samira Salihovic, Andrea Ganna, Stefan Brandmaier, Taru Tukiainen, Corey D. Broeckling, Patrik K. Magnusson, Jessica E. Prenni, Rui Wang-Sattler, Annette Peters, Konstantin Strauch, Thomas Meitinger, Vilmantas Giedraitis, Johan Ärnlöv, Christian Berne, Christian Gieger, Samuli Ripatti, Lars Lind, Nancy L. Pedersen, Johan Sundström, Erik Ingelsson, Tove Fall

**Affiliations:** 1 Department of Medical Sciences and Science for Life Laboratory, Molecular Epidemiology Unit, Uppsala University, Uppsala, Sweden; 2 Analytic and Translational Genetics Unit, Massachusetts General Hospital, Boston, MA, United States of America; 3 Program in Medical and Population Genetics, Broad Institute of MIT and Harvard, Cambridge, MA,United States of America; 4 Stanley Center for Psychiatric Research, Broad Institute of MIT and Harvard, Cambridge, MA, United States of America; 5 Department of Medical Epidemiology and Biostatistics (MEB), Karolinska Institutet, Stockholm, Sweden; 6 Research Unit of Molecular Epidemiology, Helmholtz Zentrum München, München-Neuherberg, Germany; 7 Institute of Epidemiology II, Helmholtz Zentrum München, München-Neuherberg, Germany; 8 Institute for Molecular Medicine Finland (FIMM), University of Helsinki, Helsinki, Finland; 9 Proteomics and Metabolomics Facility, Colorado State University, Fort Collins, Colorado, United States of America; 10 German Center for Diabetes Research (DZD), München-Neuherberg, Germany; 11 Department of Environmental Health, Harvard School of Public Health, Boston, Massachusetts, United States of America; 12 Institute of Genetic Epidemiology, Helmholtz Zentrum München—German Research Center for Environmental Health, Neuherberg, Germany; 13 Institute of Medical Informatics, Biometry and Epidemiology, Chair of Genetic Epidemiology, Ludwig-Maximilians-Universität, Munich, Germany; 14 Institute of Human Genetics, Helmholtz Zentrum München, Neuherberg, Germany; 15 Institute of Human Genetics, Technische Universität München, Munich, Germany; 16 Department of Public Health and Caring Sciences, Geriatrics, Uppsala University, Uppsala, Sweden; 17 School of Health and Social Studies, Dalarna University, Falun, Sweden; 18 Department of Medical Sciences, Cardiovascular Epidemiology, Uppsala University, Uppsala, Sweden; 19 Department of Medical Sciences, Clinical Diabetology and Metabolism, Uppsala University, Uppsala, Sweden; 20 Public Health, Faculty of Medicine, University of Helsinki, Helsinki, Finland; 21 Wellcome Trust Sanger Institute, Hinxton, United Kingdom; 22 Department of Medicine, Division of Cardiovascular Medicine, Stanford University School of Medicine, Stanford, CA 94305, United States of America; McGill University and Genome Quebec Innovation Centre, CANADA

## Abstract

Insulin resistance (IR) and impaired insulin secretion contribute to type 2 diabetes and cardiovascular disease. Both are associated with changes in the circulating metabolome, but causal directions have been difficult to disentangle. We combined untargeted plasma metabolomics by liquid chromatography/mass spectrometry in three non-diabetic cohorts with Mendelian Randomization (MR) analysis to obtain new insights into early metabolic alterations in IR and impaired insulin secretion. In up to 910 elderly men we found associations of 52 metabolites with hyperinsulinemic-euglycemic clamp-measured IR and/or β-cell responsiveness (disposition index) during an oral glucose tolerance test. These implicated bile acid, glycerophospholipid and caffeine metabolism for IR and fatty acid biosynthesis for impaired insulin secretion. In MR analysis in two separate cohorts (n = 2,613) followed by replication in three independent studies profiled on different metabolomics platforms (n = 7,824 / 8,961 / 8,330), we discovered and replicated causal effects of IR on lower levels of palmitoleic acid and oleic acid. A trend for a causal effect of IR on higher levels of tyrosine reached significance only in meta-analysis. In one of the largest studies combining “gold standard” measures for insulin responsiveness with non-targeted metabolomics, we found distinct metabolic profiles related to IR or impaired insulin secretion. We speculate that the causal effects on monounsaturated fatty acid levels could explain parts of the raised cardiovascular disease risk in IR that is independent of diabetes development.

## Author Summary

Impaired glucose homeostasis leads to diabetes and cardiovascular disease and has two main components: failure to secrete enough insulin from pancreatic β-cells and reduced insulin-stimulated cellular uptake of glucose and other nutrients in target tissues (insulin resistance, IR). We used metabolomics analysis in non-diabetic persons to measure a non-selective range of small molecules including amino acids, lipids, and sugars. Pathway analysis highlighted distinct metabolic pathways linked to IR (e.g. bile acid production) and impaired insulin secretion (fatty acid biosynthesis), but causal directions remained unclear. Mendelian Randomization (MR) analysis can test for causal effects in observational studies in the absence of randomized controlled trials. Using MR analysis in up to four large independent studies, we found evidence that IR causes a decrease in levels of the main endogenous monounsaturated fatty acids palmitoleic acid and oleic acid, as well as suggestive evidence for higher levels of the amino acid tyrosine. We provide a possible explanation for parts of the diabetes-independent risk of cardiovascular disease in persons with IR.

## Introduction

Insulin resistance (IR) is a major precursor of type 2 diabetes (T2D) [1], and constitutes an independent risk factor for cardiovascular disease (CVD) [2] and for certain cancer types [3, 4]. In IR, the demands on pancreatic β-cells to produce insulin increase and blood glucose levels rise if β-cell function is impaired. The metabolic effects of IR and declining β-cell function are not fully characterized and causal relationships are difficult to disentangle due to the lack of randomized controlled trials. Associations between the “gold standard” hyperinsulinemic-euglycemic clamp method [5] for measuring whole-body IR and non-targeted metabolomics profiling previously identified α-hydroxybutyrate as a biomarker for IR in 399 non-diabetic persons [6]. Additional insights for causal directions may come from profiling circulating metabolites combined with genotyping, as previously demonstrated in causal investigations of adiposity and the metabolome using a Mendelian Randomization (MR) approach [7] and for causal effects of uric acid on IR and T2D risk [8]. Mendelian randomization analysis can test the causal relationship between an exposure and an outcome variable in the absence of randomized controlled trials [9]. Exposure-associated single nucleotide polymorphisms (SNPs) can be used as instrumental variables (IVs) because allelic variants are randomly allocated at meiosis and therefore independent of bias from confounding and reverse causation. Genotype-based methods like MR can inform drug targeting: For example, the association between genetic variants in *PCSK9*, reduced low-density lipoprotein-cholesterol levels and lower coronary heart disease risk [10] predicted the clinical success of pro-protein convertase subtisilin kexin 9-inhibitors [11]. Conversely, MR studies confirmed the lack of a causal association between plasma high-density lipoprotein-cholesterol and cardiovascular events [12].

The causal effects of impaired insulin secretion and IR on blood metabolites have not been assessed before in a large-scale metabolomics framework and could pinpoint key mediators of the risk of adverse health events. We aimed to identify metabolic pathways related to IR and impaired early-phase insulin secretion during an oral glucose tolerance test (OGTT) in a large European sample and applied MR methods in additional cohorts to assess potential causal effects of impaired insulin secretion and IR. Using non-targeted metabolomics [13], we identified 52 circulating metabolites related to either IR or insulin secretion that implicated distinct metabolic pathways and we found evidence for a causal effect of IR on reduced palmitoleic acid (POA) and oleic acid (OA) levels, as well as on raised tyrosine levels.

## Results

### Metabolites associated with IR and impaired insulin secretion implicate distinct metabolic pathways

**Fig 1:**
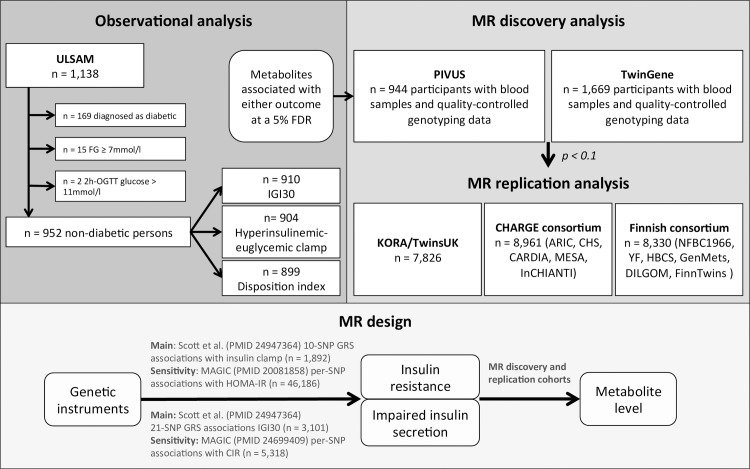
Study flow and cohorts used in the different parts of the study.

**Fig 2:**
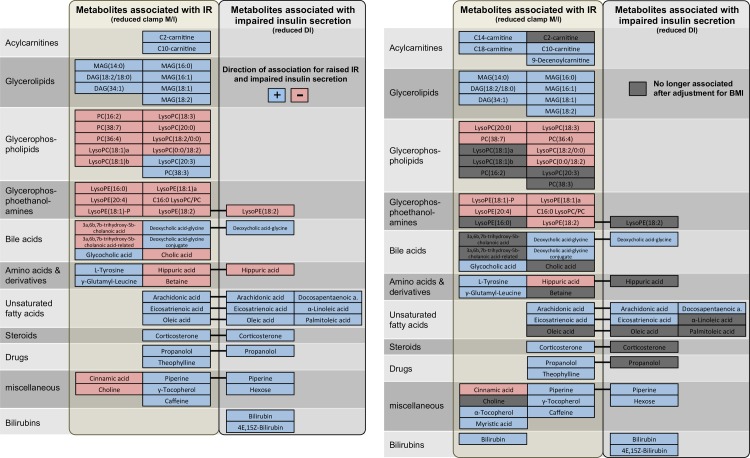
**Metabolites associated with IR and impaired insulin secretion without (left panel) and after (right panel) adjustment for BMI.** Based on linear regression models in ULSAM (n = 904 for IR, n = 899 for impaired insulin secretion) adjusted for age and sample quality; significance threshold 5% FDR.

The Uppsala Longitudinal Study of Adult Men (ULSAM) cohort of community-dwelling 71-year-old men provides the largest human sample combining plasma metabolomics with an OGTT and the hyperinsulinemic-euglycemic clamp method. We previously developed a bioinformatics pipeline for untargeted liquid chromatography/mass spectrometry (LC/MS) data [14] and were able to annotate among 10,162 spectral features 192 metabolites, on which this study is based. In up to 910 non-diabetic individuals, we used linear regression adjusted for age, sex, and sample quality indicators (S1 Text) to identify fasting metabolite levels associated with physiologic measures of insulin secretion and IR. Table 1 provides sample characteristics and Fig 1 illustrates the study flow. Three outcomes were assessed: IR (clamp M/I), the insulinogenic index (log-IGI30) as a measure of glucose-stimulated insulin secretion [15] and the disposition index (log-DI) for β-cell responsiveness [16], all scaled to SD-units. At the 5% false discovery rate (FDR), 47, 15, and zero metabolites were associated with clamp M/I, log-DI and log-IGI30, respectively (Fig 2, S1 Table). Reduced levels of lysophosphatidylethanolamine (LysoPE) 18:2 and hippuric acid were associated with IR and impaired insulin secretion. Shared positive associations were found for deoxycholic acid glycine conjugate, corticosterone, propranolol, piperine, and three unsaturated fatty acids (FAs; arachidonic, eicosatrienoic, and oleic acid). Higher levels of glycerolipids and several acylcarnitines, and lower levels of glycerophospholipids were exclusively associated with IR. Further, increased levels of two bilirubin species were exclusively associated with impaired insulin secretion. To identify metabolite associations independent of adiposity, we additionally adjusted each model for body mass index (BMI, S1 Table). This reduced the strength of associations to a lesser extent for impaired insulin secretion than for IR and preserved the direction of associations for all metabolites still significant at the 5% FDR (34 for IR and 8 for impaired insulin secretion). Six new associations were detected for IR after BMI adjustment (increased levels of three acylcarnitines, α-tocopherol, myristic acid and bilirubin, Fig 2). Pathway enrichment analysis carried out with MetaboAnalyst 3.0 [17] (http://www.metaboanalyst.ca/) indicated significant enrichment of IR-associated metabolites in primary bile acid synthesis (*p* = 0.009, 4 metabolites), glycerophospholipid metabolism (*p* = 0.006, 4 metabolites) and caffeine metabolism (*p* = 0.016, 2 metabolites) (S1 Fig). Impaired insulin secretion-associated metabolites were enriched in the FA biosynthesis pathway (*p* = 0.027, 3 metabolites).

**Table 1:** Sample characteristics. Means ± standard deviation (SD) are shown for continuous, percentages for categorical variables (characteristics of replication cohorts are detailed in S1 Text).

	ULSAM (n = 904)	PIVUS (n = 944)	TwinGene (n = 1,669)
**Age**	71.2 ± 0.5	70.2 ± 0.2	68.6 ± 8.3
**% Male**	100%	49.3%	41.1%
**Current smoker**	20.7%	10.6%	14.9%
**Anti-hypertensive medication**	31.1%	28.3%	25.2%
**Lipid-lowering medication**	8.9%	13.0%	15.1%
**BMI (kg/m**^**2**^**)**	25.9 ± 3.1	26.9 ± 4.2	26.0 ± 3.8
**Total cholesterol (mmol/l)**	5.8 ± 1.0	5.5 ± 1.0	5.8 ± 1.1
**TG (mmol/l)**	1.4 ± 0.7	1.3 ± 0.6	1.4 ± 0.8
**LDL-C (mmol/l)**	3.9 ± 0.9	3.4 ± 0.9	3.8 ± 1.0
**HDL-C (mmol/l)**	1.3 ± 0.4	1.5 ± 0.4	1.4 ± 0.4
**Waist (cm)**	94.8 ± 9.7	90.6 ± 11.4	92.9 ± 11.4
**SBP (mmHg)**	147.8 ± 18.9	149.2 ± 22.6	142.8 ± 20.5
**DBP (mmHg)**	84.3 ± 9.1	78.7 ± 9.9	82.3 ± 10.6
**hs-CRP (mg/l)**	3.2 ± 4.8	3.1 ± 4.5	3.6 ± 6.6
**Clamp M/I (mg x kg**^**-1**^ **x kg BW**^**-1**^ **x min**^**-1**^ **per mU/l x 100)**	5.4 ± 2.4	-	-
Disposition index (M/I * IGI30) ^**A**^	2.5 ± 3.3	-	-

^A^n = 899

BMI = body mass index, hs-CRP = high sensitivity C-reactive protein, DBP = diastolic blood pressure, HDL-C = high-density lipoprotein cholesterol, LDL-C = low-density lipoprotein cholesterol, SBP = systolic blood pressure, TG = triglycerides

### In MR analysis, IR lowers POA, OA and hippurate levels

#### MR analysis in the discovery set

**Fig 3:**
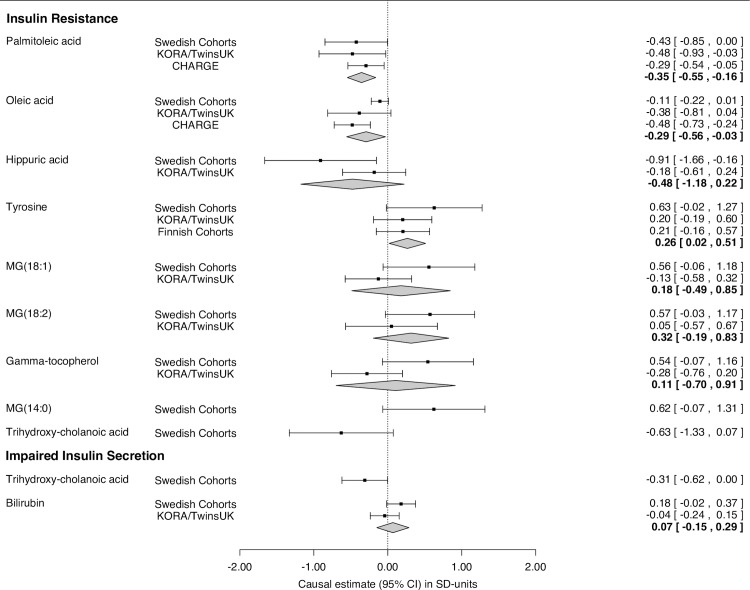
Causal estimates (95% CI) for IR and impaired insulin secretion on metabolite levels. Estimates (*p* < 0.1) in the Swedish cohorts (PIVUS and TwinGene, n = 2,613) and the replication studies (n_KORA_ = 7,824, n_CHARGE_ = 8,961, n_Finnish_ = 8,330) are expressed as SD-unit change in metabolite levels associated with a 1 SD increase in genetically determined IR (reduced clamp M/I) or impaired insulin secretion (reduced IGI30).

To examine causality in the association between insulin response and metabolite levels, we took the 52 metabolites associated with either IR or impaired insulin secretion in age- and sex-adjusted analysis forward to MR analysis in two separate Swedish cohorts—the Prospective Investigation of the Vascular in Uppsala Seniors (PIVUS) and the TwinGene study. We used the previously validated additive allelic genetic risk scores for IR (10 SNPs) and impaired insulin secretion (21 SNPs) as IVs [18] with proxies in strong linkage disequilibrium (r^2^ >0.8) in lieu of unavailable SNPs (S2 Table). Associations between genetic scores and metabolites were assessed in linear models adjusted for age, sex, the first three genetic principal components and cohort in PIVUS (n = 944) and TwinGene (n = 1,669) (Fig 1). IV-exposure associations were obtained from Scott et al. [18] and causal estimates were calculated as the ratio between the two regression coefficients (scaled to SD-units). We detected evidence of causal effects of IR on lower levels of POA (β_IV_ = –0.43, 95% CI –0.85 to –0.01, *p* = 0.048) and hippuric acid (β_IV_ = –0.91, 95% CI –1.66 to –0.16, *p* = 0.018) (S3A Table). Causal effects that approached significance (*p* < 0.1) were detected for reduced levels of OA (*p* = 0.074) and 3α,6β,7β-trihydroxy-5β-cholanoic acid (*p* = 0.079) as well as for higher levels of monoacylglycerol (MAG) 18:1 (*p* = 0.079), MAG(18:2) (*p* = 0.064), MAG(14:0) (*p* = 0.077), and γ-tocopherol (*p* = 0.084) (Fig 3 and S3 Table). Impaired insulin secretion showed evidence of a causal effect on lower levels of the C24-bile acid 3α,6β,7β-trihydroxy-5β-cholanoic acid (β_IV_ = –0.36, 95% CI –0.68 to –0.04, *p* = 0.027) and there was a trend for a level-raising effect on bilirubin (*p* = 0.071) (S3B Table).

### The negative causal effect of IR on monounsaturated FA levels is replicated in independent cohorts

We attempted replication for metabolites with suggestive evidence of causation (*p < 0*.*1*) in up to 7,824 European individuals in the KORA F4/TwinsUK cohorts that underwent untargeted metabolomics profiling on a different LC/MS platform [19]. The liberal p-value threshold was chosen as a compromise between limited sample size and the risk of missing true positive associations in the MR discovery set and we adopted the conventional threshold of *p* < 0.05 for significance in all replication analyses. Seven of the nine causally implicated metabolites for IR (excluding MAG(14:0) and 3α,6β,7β-trihydroxy-5b-cholanoic acid) and one of two (bilirubin) for impaired insulin secretion were available in KORA/TwinsUK. We replicated the causal effect of IR on POA (β_IV_ = –0.48, 95% CI –0.93 to –0.03 *p* = 0.038, all in SD-units) and the tentative effect on OA (β_IV_ = –0.38, 95% CI –0.81 to 0.04, *p* = 0.078) (Fig 3). We could not replicate the causal effects on tyrosine (β_IV_ = 0.20, 95% CI –0.19 to 0.59, *p* = 0.316) and hippuric acid (β_IV_ = –0.18, 95% CI –0.61 to 0.24, *p* = 0.398). A repeat analysis in a subset of 1,432 non-diabetic individuals in the KORA F4 cohort replicated the causal effect on POA (β_IV_ = –1.14, 95% CI –2.13 to –0.15, *p* = 0.024) and the tentative effect on OA (β_IV_ = –0.82, 95% CI –1.75 to 0.10, *p* = 0.082) (S1 Text).

In a second attempt to replicate the causal findings for POA and OA, we obtained the publicly available GWAS results for FA fractions in plasma phospholipids from the Cohorts for Heart and Aging Research in Genomic Epidemiology (CHARGE) consortium (http://www.chargeconsortium.com/main/results) [20]. The CHARGE study combines five independent cohorts of 8,961 Europeans in age, sex, and recruitment site-adjusted meta-analysis and we performed MR analysis based on the genetic IR score, as all 10 SNPs had been genotyped (S1 Text, FA fractions were converted to SD-units). The results replicated the causal effect of IR on POA (β_IV_ = –0.29, 95% CI –0.54 to –0.05, *p* = 0.018) and OA (β_IV_ = –0.48, 95% CI –0.73 to –0.24, *p* = 0.007).

We aimed to replicate the possible causal effect of IR on raised tyrosine using the summary meta-GWAS results from five cohorts of 8,330 Finnish individuals who underwent nuclear magnetic resonance metabolomics profiling [21]. In this sample, no significant effect on serum tyrosine levels was found (β = 0.21, 95% CI –0.16 to 0.57, *p* = 0.267).

Achieving adequate power in MR analysis requires large sample sizes [22]. However, the differences in analytical methods between studies for the same metabolite make the combination of within-study effects in post-hoc meta-analysis methodologically unsound. To nonetheless explore the effect of increased sample size on causal effects, we combined estimates for each metabolite in inverse variance-weighted fixed-effects meta-analysis and illustrate these exploratory estimates in Fig 3. In addition to the negative effects on POA and OA, the combined analysis indicated a causal effect of IR on higher tyrosine levels (*p* < 0.05) that was observed as a non-significant trend in each individual study. Given the risk of false positive findings due to multiple testing, these post-hoc findings should be interpreted with caution and require confirmation studies.

### MR sensitivity analysis does not indicate significant pleiotropy

To assess for violations of MR assumptions due to genetic confounding, horizontal pleiotropy and IV heterogeneity, we examined a) the association of individual SNPs with the risk factors (S2B Fig)); b) the association of IVs with potential confounders (S3C Fig)); c) scatter plots of IV-outcome v. IV-risk factor associations and funnel plots of IV strength v. IV estimate (S4D Fig)); d) implemented sensitivity analysis for individual SNPs in inverse variance-weighted, log-likelihood and MR Egger regression, including heterogeneity tests (S4 Table, S1 Text [23–25]); and e) discuss in S1 Text the likelihood of bias from canalization and other effects.

We tested for associations of genetic scores with potential mediator variables in age- and sex-adjusted linear regression in ULSAM, PIVUS and TwinGene (S3 Fig) and confirmed the association between worse genetic IR and lower HDL-C as well as smaller waist-hip ratio as reported by Scott et al. [18] (who included ULSAM), and found an association with higher albumin levels. As in [18], the impaired insulin secretion score was positively associated with plasma glucose and unrelated to other traits apart from lower albumin levels and a trend for increased C-reactive protein. For all metabolites with indication for a causal effect (*p* < 0.1) in the MR discovery sample, we examined individual SNP effects on metabolite levels. The IV-exposure associations were derived from the GWAS results for homeostasis model assessment-IR (HOMA-IR, n = 46,186) and corrected insulin response (CIR, n = 5,318) in non-diabetic persons from the publicly available results of the Meta-Analyses of Glucose and Insulin-related traits Consortium (MAGIC; http://www.magicinvestigators.org/downloads/, converted to SD-units). The association between SNPs and risk factors in MAGIC were consistent and did not indicate heterogeneity apart from one variant (rs11605924) that was associated with CIR in the unexpected direction (S2 Fig). As reported in S4 Table, Cochran’s *Q* tests failed to detect significant heterogeneity between individual SNPs’ causal estimates. The significant effects of IR on OA and POA levels detected in the main analysis were replicated in all sensitivity tests except in the case of MR Egger regression for POA (slope estimate –0.96, 95% CI –2.15 to 0.22, *p* = 0.095). Although the intercept estimate in MR Egger regression for OA differed significantly from zero, it was small in magnitude (intercept estimate 0.02, 95% CI 0.00 to 0.04, *p* = 0.018) and the causal effect was reproduced (slope estimate –0.49, 95% CI –0.82 to –0.16, *p* = 0.010). One important assumption of MR Egger regression–that any pleiotropic effects of IVs on the outcome be independent of IV strength [23]–cannot be assessed with currently available methods. Because the IV score had been carefully constructed by Scott et al. [18] to limit the likelihood of pleiotropic interference (particularly from BMI) and based on the totality of all MR sensitivity analyses that reproduced the main results in direction and magnitude and failed to indicate significant heterogeneity, we are confident to have excluded pleiotropic effects that could invalidate our main findings as far as possible. However, as discussed in S1 Text, some sources of bias (e.g., canalization) cannot be excluded and the possibility of remaining pleiotropic effects pose limitations that mandate careful interpretation of any MR study.

### Publicly available gene expression data support the causal effects on OA and POA

To further assess our findings of a negative causal effect of IR on monounsaturated FA levels, we looked up gene expression data for *SCD1* and its rodent equivalent *scd1* in the EMBL-EBI Expression Atlas v3.0 (http://www.ebi.ac.uk/gxa/home). This gene encodes stearoyl-CoA desaturase 1 (SCD-1), the rate-limiting enzyme in the biosynthesis of OA and POA [26]. Among 252 uploaded experiments that reported significantly different expression (5% FDR) between experimental and control conditions, we extracted all experiments related to IR. There were eight studies in mice and rats–none in human beings (S1 Text). In all instances, the direction of differential *scd1* expression was consistent with our findings—the IR-increasing condition down-regulated *scd1* expression.

## Discussion

In the observational part of this multi-cohort study of blood metabolomics profiles, we identified bile acid, glycerophospholipid and caffeine metabolism as associated with IR, and FA biosynthesis as related to impaired insulin secretion. We discovered and replicated causal effects of IR on lower levels of the monounsaturated FAs POA and OA, as well as suggestive evidence for higher levels of the aromatic amino acid tyrosine. Sensitivity analyses did not indicate pleiotropic effects of the genetic instruments. Causal effects were largely unaffected by the exclusion of prevalent diabetes cases in the KORA/TwinsUK replication set. A small collection of publicly available experimental results in rodents supported our causal findings: All IR-increasing conditions were associated with a down-regulation of SCD-1, implying reduced endogenous production of OA and POA.

The liver and adipose tissue are the main sites of de novo lipogenesis and SCD-1 is the rate-limiting enzyme for monounsaturated FA biosynthesis [27]. It introduces double bonds into palmitic and stearic acid to produce POA (16:1n-7) and OA (18:1n-9), respectively–the major precursors for cholesteryl esters and triglycerides (TGs) that are packaged into very low-density lipoprotein (VLDL) particles and secreted by the liver [28]. In *scd1* knockout mice and hypertriglyceridemic persons, plasma FA composition reflects hepatic SCD-1 activity [29, 30]. Hence, whilst dietary FAs contribute to overall plasma FA levels, the relative lipid composition, as assessed in the present study, is likely to reflect SCD-1 activity. Correspondingly, in ULSAM and PIVUS, we found good agreement between FA quantification by untargeted plasma metabolomics and targeted serum cholesteryl ester analysis (S1 Text).

Experimental evidence for the inhibition of SCD-1 by IR stems from liver-specific insulin receptor knockout (LIRKO) mice that had ~80% reduced hepatic *scd1* expression and ~90% reduced microsomal *scd1* transcript levels compared to control mice [31, 32]. In muscle-specific insulin receptor knockout (MIRKO) mice, *scd1* expression was downregulated by ~23% compared to controls [33].

**Fig 4:**
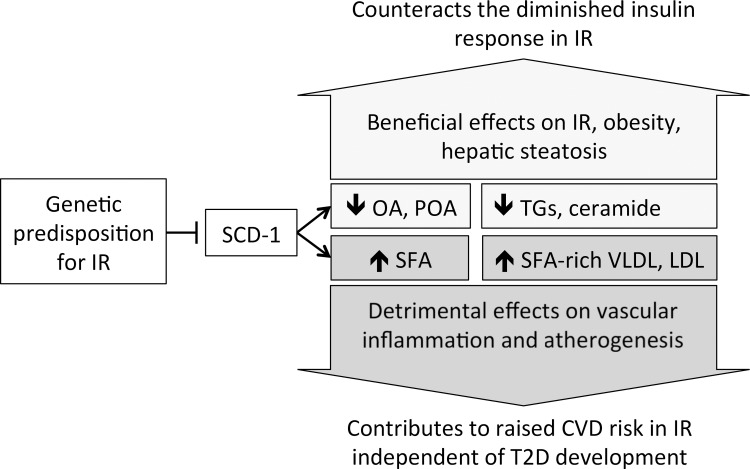
Schematic summary of the presumed effects of IR on SCD-1 and associated consequences. Abbreviations: IR–insulin resistance, LDL–low density lipoprotein, OA–oleic acid, POA–palmitoleic acid, SCD-1 –stearoyl CoA-desaturase 1, SFA–saturated fatty acid, TG—triglycerides, VLDL–very low density lipoprotein

Little is known about the causal effects of a genetic predisposition for IR on SCD-1 [34]. Reduced SCD-1 activity in knockout mice has beneficial metabolic consequences, including reduced obesity [35] and improved IR [36] (reviewed in [28] and [34]). Yet, reduced SCD-1 activity has also been associated with adverse vascular outcomes: Inhibition of SCD-1 in hyperlipidemic mice markedly increased aortic atherosclerosis despite protective effects on obesity and IR [37]. Reduced SCD-1 activity caused enrichment of saturated FA in VLDL and LDL, which promoted atherogenesis through macrophage-induced vascular inflammation. Susceptibility to exacerbated inflammation was also demonstrated in *scd1* knockout mice with induced colitis [38] or on a very low-fat diet that increased endoplasmic reticulum stress response [39]. Based on these competing effects on vascular and metabolic health, we speculate that IR reduces SCD-1 activity, which counteracts the metabolic consequences of IR by improving insulin signaling but concomitantly increases the risk for CVD through saturated FA-induced proinflammatory changes. Supported by our results and the above evidence, this hypothesis further derives from two facts: IR increases CVD risk independent of other risk factors [2, 40] and IR predicts CVD risk independent of T2D [41]. A summary of the presumed relationships is displayed in Fig 4. In our study, we could not evaluate the longitudinal effect on CVD, hence our hypothesis needs to be evaluated in cohorts with the available outcome data. In observational analysis, IR was positively associated with OA (FDR-adjusted *p* = 0.035, after adjustment for BMI *p*_*BMI*_ > 0.05) and POA levels (*p* = 0.091, *p*_*BMI*_ > 0.1) in contrast to the negative genetic associations in MR studies. This discrepancy is likely due to confounding factors such as dietary FA intake and highlights that MR studies have the power to disentangle causal mechanisms that may be obscured in cross-sectional studies. The hypothesized but untested relationship with cardiovascular disease requires investigation in future studies.

The effect of IR on elevated tyrosine levels was observed as a non-significant tendency in all cohorts but reached nominal significance only after combination of estimates in meta-analysis. Although there is a considerable risk that this result is a false positive due to multiple testing, we still consider it an interesting finding worth investigating in larger MR studies, as it has not been reported before. Associations between worse IR/T2D risk and circulating tyrosine levels have been established in observational and longitudinal studies [42, 43]. Possible molecular mechanisms could involve reduced tyrosine catabolism, either through IR-induced oxidative stress leading to elevations in methionine, cysteine and the antioxidant glutathione that result in tyrosine hydroxylase inhibition [44]; or through inactivation of tyrosine aminotransferase [45]. Hence, we speculate that the previously identified association between tyrosine and increased T2D risk could be caused by concomitant IR but caution that the observed trends in our study need to be verified in larger samples.

Limitations of our study include moderate power in MR studies and inherent limitations of the non-targeted metabolomics discovery platform to capture certain metabolites (e.g., polar amino acids) and separate sugars and other non-polar molecules. We used an untargeted metabolomics approach that detected several thousand metabolic features, yet the limited availability of standard compound spectra in in-house and public libraries precluded the quantification of the entire spectrum of the plasma metabolome and may have biased the pathway enrichment analysis. Observational associations were established in an exclusively male, elderly European cohort (ULSAM) with unknown generalizability to other age and ethnic groups. The different metabolomics platforms between studies and the expression of FA levels in % of plasma phospholipids rather than absolute levels in CHARGE somewhat limit methodological consistency. However, this diversity also supports the robustness of findings that were replicated on different platforms. Causal relationships were consistent across studies but require validation in physiologic models of IR, particularly as some sources of bias (e.g., canalization, unmeasured horizontal pleiotropy) cannot be excluded. Whilst the unique contribution of our study is to assess causal effects on the plasma metabolome, we could not examine the reverse causality as no genetic instruments were available for the majority of metabolites. As illustrated above, relative levels of circulating POA and OA likely reflect endogenous biosynthesis, however, the exact contributions of SCD-1, exogenous sources, catabolism and excretion could not be addressed by our study and require experimental investigation. We used proxy outcomes (HOMA-IR and CIR) with available large GWAS results in sensitivity analyses rather than the exact same measures as in the main analysis.

Strengths include the extensive testing for violations of MR assumptions, the replication of main findings in large independent cohorts that used different methods of quantification, and the use gold standard measures for IR. We detected no evidence of pleiotropy of genetic instruments whose associations with cardiometabolic traits were in the expected directions and causal estimates agreed between different analysis methods.

In summary, our study in multiple independent cohorts of community residents indicates a causal effect of IR on circulating OA, POA, and tyrosine levels and provides new insights into the metabolomic signature of IR and impaired insulin secretion. It is to our knowledge the first large-scale attempt to explore the causal effects of genetic IR on a non-selected set of plasma metabolites. The potential implications of the presumed IR-induced inhibition of monounsaturated FA biosynthesis on health outcomes require validation in experimental models but may form part of the explanation for the elevated CVD risk in IR that is independent of T2D development.

## Materials and Methods

### Cohort description

Detailed descriptions are available in S1 Text. In brief, ULSAM (http://www2.pubcare.uu.se/ULSAM/) was started in 1970 and enrolled 81.7% (n = 2,322) of all male residents of Uppsala county, Sweden, born between 1920 and 1924. On-going assessments every five to ten years include questionnaire, biochemical, and anthropometric examinations. The current study is based on the assessment at age 70 years, which included an OGTT and hyperinsulinemic-euglycemic clamp measurement.

The PIVUS study (http://www.medsci.uu.se/pivus/) enrolled 50% (n = 1,016) of a random sample of Uppsala community residents aged 70 years in 2001 and features assessments including health questionnaires, blood sampling and clinical measurements every five years. The current study is based on the assessment at age 70 years.

TwinGene (http://ki.se/en/meb/twingene-and-genomeeutwin) is a longitudinal study of 12,591 twins born before 1958 and registered in the Swedish Twin Registry with questionnaire assessments and blood sampling done between 1998 and 2002, and again between 2004 and 2008. The current study used a subcohort from a nested case-cohort design established for an earlier project [13] that was randomly selected within four age and sex strata to match a case group that included incident cases of T2D, coronary heart disease, ischemic stroke, and dementia (up to 31 Dec 2010) (S1 Text). In all three Swedish cohorts, prevalent cases of diabetes were excluded (criteria in S1 Text).

The Cooperative Health Research in the Region of Augsburg (*Kooperative Gesundheitsforschung in der Region Augsburg*, KORA [46]) study is a series of epidemiological surveys of the general population in Southern Germany that includes longitudinal health assessment and blood sample collection. The current study is based on the KORA F4 (2006–2008) survey of 32-to-77-year-old men and women (n = 1,768, 48.5% male, mean age 60.8 ± 8.8 years, mean BMI 28.2 ± 4.8 kg/m^2^).

TwinsUK is a predominantly female cohort of adult twins recruited from the general UK population. The current study is based on 17-to-85-year-old twins (n = 6,056, 7.1% male, mean age 53.4 ± 14.0 years, mean BMI 26.1 ± 4.9 kg/m^2^) who underwent blood metabolite profiling and health center assessment.

The CHARGE consortium GWAS for plasma FA fractions [20] combined 8,961 mostly middle-aged to older persons of European ancestry (45.0% male, mean age 59.7 ± 7.6 years, mean BMI 27.0 ± 4.8 kg/m^2^) from five cohorts—the Atherosclerosis Risk in Communities (ARIC) study, the Cardiovascular Health Study (CHS), the Coronary Artery Risk Development in Young Adults (CARDIA) study, the Invecchiare in Chianti (InCHIANTI) study, and the Multi-Ethnic Study of Atherosclerosis (MESA). Details on cohorts and recruitment are reported elsewhere [20] and documented online (http://chargeconsortium.com/).

The Finnish consortium [21] combined five cohorts of 8,330 individuals with serum nuclear magnetic resonance metabolomics results from the FINRISK 2007 Dietary, Lifestyle and Genetic determinants of Obesity and Metabolic syndrome (FINRISK-07/DILGOM) study, the Helsinki Birth Cohort Study (HBCS), the Health2000 GenMets study, the Northern Finland Birth Cohort 1966 (NFBC1966) study and the Cardiovascular Risk in Young Finns Study (YF). Across all cohorts with 46.9% males, mean age was 37.8 ± 3.2 years and mean was BMI 25.4 ± 4.3 kg/m^2^.

### Ethics statements

All participants provided written informed consent prior to inclusion in the study and the research was approved by the Ethics Committees of Uppsala University (ULSAM, PIVUS) and Karolinska Institutet (TwinGene), or the respective Institutional Review Boards for the other cohorts. The study was conducted according to the principles of the Declaration of Helsinki.

### Metabolomics profiling

Untargeted metabolomics profiling of venous blood samples in the three Swedish cohorts was carried out by ultra-performance liquid chromatography (UPLC) on a Waters Acquity UPLC system coupled to a Waters Xevo G2-Time-Of-Flight-Mass Spectrometry (TOFMS) platform at Colorado State University (Fort Collins, CO, USA). Data acquisition in the positive electrospray ion mode with a mass-to-charge ratio (m/z) range of 50–1,200 at 5 Hz was alternately performed at collision energies of 6V and 15–30V. Details on sample handling and data processing by XCMS in R [47] are available in S1 Text and in [14]. Parameter selection for feature detection, alignment, grouping, and imputation was optimized in simulations of random sets of 20–40 samples. In total, 10,162 (ULSAM), 9,755 (TwinGene) and 7,522 (PIVUS) features were detected. Adjustment for factors of unwanted variability (plate effect, analysis date, retention time drift and sample collection) by analysis of variance-type standardization was followed by log-transformation and removal of spectra with abnormal intensities and/or low inter-duplicate correlations and/or retention times <35 sec. For each feature, retention time, m/z, and fragmentation pattern were compared to in-house and public database reference libraries and matched according to Metabolomics Standard Initiative guidelines [48]. The current study is based on all 192 metabolites identified in ULSAM. Common features between ULSAM, PIVUS, and TwinGene were identified by matching m/z and retention time, followed by manual inspection of fragmentation spectra. Full metabolomics data are available in the MetaboLights archive (study identifiers MTBLS90 for PIVUS, MTBLS124 for ULSAM, MTBLS93 for TwinGene; http://www.ebi.ac.uk/metabolights/).

Metabolomics analyses in KORA and TwinsUK were carried out by the commercial company Metabolon, Inc. (Durham, NC, USA), which combined positive and negative ion-mode UPLC/tandem-MS with gas chromatography/MS. Following protein precipitation in methanol, samples for analyzed in duplicates and spectral annotation was performed against a standard compound library (for details, see S1 Text and [19, 46]). Following the removal of outlying (>3 SD) features and those with <300 non-missing values, 276 and 258 metabolites in KORA and TwinsUK, respectively, were quantified and identified based on matching to in-house library standards.

In CHARGE, a targeted gas chromatography approach was used to quantify plasma phospholipid composition (except in the InCHIANTI cohort, where total plasma FA were measured). As detailed in [20], fasting plasma phospholipid isolation by thin-layer chromatography was followed by quantification of FA by targeted gas chromatography.

The Finnish study processed serum samples from all five sub-cohorts in one central laboratory by three complementary ^1^H-nuclear magnetic resonance analysis windows optimized for lipoproteins, low molecular weight metabolites and lipid species, respectively [21, 49]. For quantitative analysis, raw spectral data were pre-processed with baseline zeroing, peak alignment and correction for albumin background and validated against high-performance LC data.

### Statistical analysis

#### Outcome measures in ULSAM

The outcomes were insulin sensitivity (clamp M/I in mg x kg^-1^ x kg body weight^-1^ x min^-1^ per mU / l x 100; representing the amount of glucose metabolized per unit of plasma insulin during the clamp assessment), the insulinogenic index (IGI30) as a measure of glucose-stimulated insulin secretion [15] (calculated as the increment in plasma insulin divided by the change in plasma glucose levels between 0–30 min during the OGTT, log-transformed to normality), and the disposition index (DI) for β-cell responsiveness [16] (the product of clamp M/I x IGI30, log-transformed to normality). All outcomes were scaled to SD-units prior to analysis.

#### Association testing in ULSAM

Details on the OGTT and hyperinsulinemic-euglycemic clamp testing (modified according to [5]) are available in S1 Text. Separate multivariable age-adjusted linear regression analyses with or without additional adjustment for BMI were carried out for blood metabolite levels (standardized to mean = 0, SD = 1) as predictors for clamp M/I, IGI30 [15] and DI [16]. Non-normal distribution according to histograms required natural log-transformation for IGI30 and DI. Model assumptions were ascertained in residual-by-fitted value plots and outliers screened for in histograms and plots of Cook’s distance. Metabolites with significant associations after adjustment for multiple testing at the 5% FDR were taken forward to MR analysis. All analyses were performed in R v3.1.1. To ease understanding, in the manuscript all associations are expressed in the direction of worsening IR and impaired insulin secretion (except in S1 Table where associations with the actual measure–clamp M/I, DI, and IGI30 –are presented).

### MR analysis

We used IV analysis to estimate the causal effect of IR/impaired insulin secretion on metabolite levels in PIVUS and TwinGene. The Wald ratio estimator [50] was obtained as the ratio between the regression coefficients for the effect of the genetic IV on metabolite levels divided by the effect of the genetic IV on IR/insulin secretion (Eq 1). Standard errors were estimated by the *delta* method, which we previously validated (Eq 2) [51].

$$\beta_{IV}=\frac{\beta_{IV-Metabolite}}{\beta_{IV-Exposure}}$$

$SE_{IV}=ABS(\beta_{IV})\sqrt{{\left(\frac{SE_{IV-Metabolite}}{\beta_{IV-Metabolite}}\right)}^{2}+{\left(\frac{SE_{IV-Exposure}}{\beta_{IV-Exposure}}\right)}^{2}}$**Eq 1 and 2.** Causal estimator (β_IV_) and standard error (SE_IV_) calculations based on regression coefficients and corresponding SEs for the IV in linear models with exposure or metabolite levels as outcome.

As IV, we used the IR and impaired insulin secretion genetic risk scores validated by Scott et al. [18] (S2 Table). Because the study included ULSAM (alongside the MRC-Ely, RISC, Fenland, and EPIC-Interact cohorts) to estimate the association between instrument and risk factor, we excluded the ULSAM cohort from MR analysis. We used proxy SNPs (linkage disequilibium r^2^ >0.8) for variants not directly genotyped. When no proxies were available, SNP scores were imputed as 2*risk allele frequency based on the 1000 Genomes Project phase 1 [52] (for the insulin secretion score this applied to rs1800574 in all three cohorts, as well as rs7957197 and rs10811661 in TwinGene) (S2 Table). Quality control included mean-imputation of SNP values for individuals with one missing value and exclusion of individuals with >1 missing values. All SNPs had call rates >95%. Additive non-weighted genetic risk scores were calculated in PLINK1.07 (http://pngu.mgh.harvard.edu/~purcell/plink/). The association between genetic IVs and metabolites was estimated in linear models adjusted for age, sex, the first three genetic principal components and cohort with metabolite levels (SD-unit) as outcome. Age- and sex-adjusted associations between genetic IVs and exposure (SD-unit) were obtained from Scott et al. [18]. For sensitivity analysis, SNP associations with IR and insulin secretion were obtained from summary GWAS results in MAGIC (see above). MR Egger regression and other sensitivity analyses were carried out in R according to the scripts provided in the data supplement by [23] and in appendix A.3 by [24], respectively.

### Replication in KORA/TwinsUK, CHARGE, and the Finnish cohorts

We obtained the publicly available GWAS data for serum/plasma metabolite levels in 7,824 European adults in the KORA F4 and TwinsUK cohorts [19], as well as for OA and POA fractions of total plasma phospholipids in 8,961 Europeans in CHARGE http://chargeconsortium.com/). We also obtained the summary meta-GWAS statistics for serum tyrosine levels from a Finnish consortium study [21]. Details on genotyping and statistical analyses are available in S1 Text and elsewhere [19, 46]. We extracted β coefficients and standard errors for SNPs in the non-weighted IR/insulin secretion genetic scores and computed summary effect sizes with the *grs*.*summary()* function in the *gtx* package in R [53]. Regression coefficients expressed SD-unit change in metabolite levels. Causal effects were estimated by MR analysis as described above for all metabolites available in KORA/TwinsUK that passed *p < 0*.*1* at the discovery stage, for OA and POA in CHARGE, and for tyrosine in the Finnish consortium. Causal estimate from all cohorts were combined in inverse variance-weighted, fixed effects meta-analysis via *metafor* in R.

R scripts for the full metabolomics pipeline in PIVUS, TwinGene and ULSAM are available online (https://github.com/andgan/metabolomics_pipeline), R scripts used for observational and MR analysis are available as well (https://github.com/chrnowak/metabolomics).

## Supporting Information

S1 Text Supplemental methods and results. (DOCX)

S2 Text Product ion spectra from liquid chromatography/tandem mass spectrometry analysis of selected metabolites and their corresponding standards that were used for annotation in ULSAM, PIVUS, and TwinGene. (DOCX)

S1 Fig Metabolic pathway enrichment for 47 IR-associated metabolites and 15 impaired insulin secretion-associated metabolites in observational analysis. (TIF)

S2 Fig Associations between genetic risk scores for IR (S2A Fig) and impaired insulin secretion (S2B Fig) with HOMA-IR and CIR, respectively, in MAGIC. (TIF)

S3 Fig **Associations between genetic risk scores for IR (upper panel) and insulin secretion (lower panel) with cardiometabolic traits in ULSAM, PIVUS and TwinGene.** Based on linear regression adjusted for age, sex, and cohort. Beta coefficients and 95% CIs indicate the SD-unit change in trait level per added risk allele. ALP = alkaline phosphatase, ALT = alanine amino transferase, ApoA1/B = apolipoprotein A1/B, BMI = body mass index, CK = creatinine kinase, CRP = C-reactive protein, DBP = diastolic blood pressure, Hb = hemoglobin, HDL-chol = high-density lipoprotein cholesterol, IGI30 = insulinogenic index, IL-6 = interleukin-6, LDL-chol = low-density lipoprotein cholesterol, SBP = systolic blood pressure, TG = triglycerides, WHR = waist-hip ratio
(TIF)

S4 Fig MR sensitivity plots for top metabolites in all cohorts: scatter plots of genetic associations with the outcome against genetic associations with the risk factor, and funnel plots of IV strength against IV estimate. (PDF)

S1 Table Associations between clamp M/I, IGI30, and DI with fasting metabolite levels in ULSAM. (XLSX)

S2 Table Genetic score SNPs and availability in the discovery cohorts. (XLSX)

S3 Table MR analysis results for causal effects of IR and impaired insulin secretion on metabolite levels in the discovery cohorts. (XLSX)

S4 Table Results of MR sensitivity analysis for top metabolites. (XLSX)

S5 Table Metabolic features, annotation levels and main adduct form in liquid chromatography/tandem mass spectrometry analysis in ULSAM, PIVUS, and TwinGene. (XLSX)
