# Correction: Effect of Insulin Resistance on Monounsaturated Fatty Acid Levels: A Multi-cohort Non-targeted Metabolomics and Mendelian Randomization Study

**DOI:** 10.1371/journal.pgen.1007002

**Published:** 2017-09-14

**Authors:** Christoph Nowak, Samira Salihovic, Andrea Ganna, Stefan Brandmaier, Taru Tukiainen, Corey D. Broeckling, Patrik K. Magnusson, Jessica E. Prenni, Rui Wang-Sattler, Annette Peters, Konstantin Strauch, Thomas Meitinger, Vilmantas Giedraitis, Johan Ärnlöv, Christian Berne, Christian Gieger, Samuli Ripatti, Lars Lind, Nancy L. Pedersen, Johan Sundström, Erik Ingelsson, Tove Fall

Panel 1 of S2 Text shows the wrong metabolic feature for oleic acid. Please view the correct S2 Text below.

## Supporting information

S2 Text Product ion spectra from liquid chromatography/tandem mass spectrometry analysis of selected metabolites and their corresponding standards that were used for annotation in ULSAM, PIVUS, and TwinGene. (DOCX)
