# Supplementary material for: Effect of Insulin Resistance on Monounsaturated Fatty Acid Levels: A Multi-cohort Non-targeted Metabolomics and Mendelian Randomization Study
Source: PLoS Genet. 2016 Oct 21;12(10):e1006379. doi: 10.1371/journal.pgen.1006379 (PMC5074591; doi:10.1371/journal.pgen.1006379)
Supplement: S1 Text — (DOCX) [file pgen.1006379.s001.docx]

Nowak et al. Effect of insulin resistance on monounsaturated fatty acid levels: a multi-cohort non-targeted metabolomics and Mendelian randomization study

**S1 Text: Supplemental methods and results**

1. **Adjustment for blood sample quality in ULSAM**
2. **Cohort descriptions**
3. **Oral glucose tolerance test and hyperinsulinemic-euglycemic clamp testing in ULSAM**
4. **Genotyping, imputation, and statistical analysis**
5. **Metabolomics profiling in ULSAM, PIVUS and TwinGene**
6. **Metabolomics profiling in the replication cohorts KORA/TwinsUK, CHARGE and the Finnish consortium**
7. **Assessment of MR analysis limitations**
8. **Power calculation for MR analysis**
9. **MR results and demographics in KORA F4 non-diabetic persons**
10. **Gene expression look-up for *scd1***
11. **Comparison between whole plasma metabolomics and serum cholesteryl ester fatty acid analysis in ULSAM and PIVUS**

**1) Adjustment for blood sample quality in ULSAM**

In linear regression models, we adjusted for sample quality indicators as sources of unwanted variability. Information on quality was extracted from logbooks maintained by laboratory staff and cohort administrators. Assessment time as a continuous variable in days between first (day 0) and last (day 749) date of blood sampling was included to control for possible variation due to drift in laboratory practice and assay results. Three quality indicator variables were adjusted for: previous thawing and re-freezing (26.0% of samples), sample type (0.3% citrated plasma, 99.7% EDTA plasma), possible hemolysis (2.7%).

**2) Cohort descriptions**

***TwinGene.*** The TwinGene project (<http://ki.se/en/meb/twingene-and-genomeeutwin>) is a longitudinal cohort of 12,591 persons listed in the Swedish Twin Registry that is maintained by Karolinska Institutet in Stockholm to examine associations between genetic data and cardiovascular outcomes [1]. Swedish twins born before 1958 were assessed twice – between 1998 and 2002, and for follow-up between 2004 and 2008 – by telephone interview to administer lifestyle and health questionnaires, as well as in local health centers for blood sampling and anthropometric measurements. The current study is based on a case-cohort design described in detail elsewhere [2], in which we selected all incident cases of type 2 diabetes, coronary heart disease, ischemic stroke and dementia up to 31^st^ Dec 2010 and a control cohort of 1,643 persons without incident events. The subgroup of cases was stratified on sex and median age and a random selection of control persons proportional to the number of cases in the four strata was obtained. Blood samples were stored on ice and sent by overnight shipment to Karolinska Institutet where they were stored until analysis at -80° C. We excluded diabetic persons, defined as fasting blood glucose ≥7 mmol/l, HbA1c ≥6.5% (48 mmol/mol), a registered (according to the Swedish Hospital Discharge Register) or self-reported diagnosis of diabetes, and/or the use of anti-diabetic medication according to the Swedish Prescribed Drug Register ATC code A10.

***ULSAM.*** The ongoing Uppsala Longitudinal Study of Adult Men born between 1920-1924 (ULSAM [3]) was initiated in 1970 as a community cohort (*n* = 2,322 or 81.7% of eligible residents enrolled). Participants are assessed with a battery of questionnaire, anthropometric, and biochemical assessments every five to ten years and records are linked to the Swedish healthcare, death and drug prescription registers. The current study uses data and blood samples obtained at age 70 years (*n* = 1,221 or 72.6% of 1,681 men still alive recruited). Participants who underwent an oral glucose tolerance test (OGTT), hyperinsulinemic-euglycemic clamp assessment, and blood sampling (stored at -80° C) with subsequent metabolomics profiling were eligible for participation. We excluded diabetic persons, defined as fasting blood glucose ≥7 mmol/l, a 2h-OGTT blood glucose >11 mmol/l, a registered diagnosis of diabetes in the Swedish Hospital Discharge Register, and/or the use of anti-diabetic medication according to the Swedish Prescribed Drug Register ATC code A10.

***PIVUS.*** The Prospective Investigation of the Vasculature in Uppsala Seniors (PIVUS) study (<http://www.medsci.uu.se/pivus/> [4]) was started in 2001 as a community-based study inviting a random sample 2,025 men and women aged 70 years and living in Uppsala, Sweden, to participate in an evaluation of endothelial function (n = 1,016 enrolled, 50% female). Assessments, including health questionnaires, blood sampling and routine clinical measurements, are carried out every five or ten years. Following centrifugation, fasting plasma samples were frozen and stored at -80° C until analysis. We excluded diabetic persons, defined as fasting blood glucose ≥7 mmol/l, a registered diagnosis of diabetes according to the Swedish Hospital Discharge Register, and/or the use of anti-diabetic medication according to the Swedish Prescribed Drug Register ATC code A10.

***KORA*.** The current study is based on the KORA S4 survey, which includes 1,768 community residents (aged 32-81 years) of the city of Augsburg, Germany, who, as part of the KORA series in population-based surveys, underwent health assessment and blood sampling between 2006-2008 [5]. Morning blood samples were obtained after a minimum of 10 h overnight fast, centrifuged, and stored at -80 °C until analysis.

***TwinsUK*** was established as a UK adult twin registry cohort. The current study is based on an unselected sample (aged 32-77 years) of predominantly female twins recruited from the general population through a media campaign (n = 6,056) [6]. Blood samples were obtained after at least 6 h of fasting, centrifuged, and stored at -45 °C until analysis.

***CHARGE.*** The Cohorts for Heart and Aging Research in Genomic Epidemiology (CHARGE) consortium combined five cohorts for a genome-wide association study (GWAS) of plasma lipid fractions (<http://www.chargeconsortium.com/main/results> [7]). These were the Atherosclerosis Risk in Communities (ARIC, n = 3,269) study, the Cardiovascular Health Study (CHS, n = 1,507), the Coronary Artery Risk Development in Young Adults (CARDIA, n = 2,404) cohort, the Invecciare in Chianti (InCHIANTI, n = 1,075) study, and the Multi-Ethnic Study of Atherosclerosis (MESA, n = 706) study.

***The Finnish cohorts*.** The Finnish meta-GWAS [8] combined five cohorts of 8,330 middle-aged unrelated individuals: the FINRISK 2007 Dietary, Lifestyle and Genetic determinants of Obesity and Metabolic syndrome study (FINRISK-07/DILGOM, n = 443), the Helsinki Birth Cohort Study (HBCS, n = 708), the Health2000 GenMets study (n = 572), the Northern Finland Birth Cohort 1966 (NFBC1966, n = 4,703) study and the Cardiovascular Risk in Young Finns Study (YF, n = 1,904).

**3) Oral glucose tolerance test and hyperinsulinemic-euglycemic clamp testing in ULSAM**

In ULSAM, an OGTT was performed in overnight fasted individuals. Following baseline blood sampling, participants ingested 75 g of glucose in 300 ml of water with repeated blood sampling over the ensuing 120 min. A modified hyperinsulinemic-euglycemic clamp according to the original protocol by DeFronzo and colleagues [9], with a slightly higher insulin infusion rate than originally proposed to better suppress hepatic glucose output, was performed on a separate day. Briefly, after placement on a warmed blanket, the preferred forearm was used for intravenous cannulation in the antecubital fossa (infusions) and dorsum of the hand (blood sampling). 40 min after cannulation, a baseline blood sample was taken and a bolus dose of semisynthetic human insulin was given over 10 min followed by infusion at a rate of 56 mU x min^-1^ x m^2^ body surface area^-1^ for 110 min to achieve a steady state of hyperinsulinemia. The target plasma glucose level of 5.1 mmol/l was maintained through a 20% glucose infusion titrated in reference to plasma glucose measurements taken every 5 min (measured in duplicates with a Glucose Analyze). Steady-state plasma glucose and plasma insulin concentrations were obtained from the mean of values obtained between 60-120 min. The insulin sensitivity index (clamp M/I in mg x kg^-1^ x kg body weight^-1^ x min^-1^ per mU / l x 100) was calculated by dividing the glucose disposal index M (the amount of glucose taken up between 60-120 min in mg x kg^-1^ x kg body weight^-1^) by the mean insulin concentration during the corresponding period. Details of other investigations can be found online (<http://www2.pubcare.uu.se/ULSAM/invest/70yrs/meth70.htm#06>).

**4) Genotyping, imputation, and statistical analysis**

Because the ULSAM cohort was used by Scott et al. [10] to validate the genetic risk score, we did not include it in MR analysis but still report details on genotyping (which was similar to PIVUS) below.

***ULSAM, PIVUS, TwinGene.*** Genotyping in PIVUS and TwinGene was performed on the Illumina Human OmniExpress (ca. 700,000 SNPs); ULSAM was genotyped on the Illumina Human Omni2.5M (ca. 2,500,000 SNPs); PIVUS and ULSAM were additionally genotyped on the Illumina CardioMetabochip array. Genotyping was carried out with the Illumina GoldenGate assay system on Illumina BeadStation 500GX equipment at the Uppsala University SNP Technology Platform. Quality control in all three studies demanded minor allele frequency (MAF) >3%, *P*(Hardy-Weinberg) >10^-6^, SNP call rate >95%, genotype call rate per individual >97%, equality between reported and X chromosome-identified sex, and Info score >0.4. Imputation to the 1000G CEU (Phase 1, v.3) reference panel was performed in IMPUTE2 (<http://mathgen.stats.ox.ac.uk/impute/impute_v2.html>). Unweighted additive genetic risk scores based on variants reported by Scott et al. [10] were computed in PLINK (<http://pngu.mgh.harvard.edu/~purcell/plink/>). Further details on genotyping have been reported elsewhere [11-13].

***KORA/TwinsUK.*** Detailed descriptions of GWAS methods in KORA and TwinsUK have been reported previously [5, 6, 14]. In KORA F4, genotyping was performed on the Affymetrix GeneChip 6.0 and genotypes determined via the Birdseed2 clustering routine. Quality control mandated a SNP call rate >95% and *P*(Hardy-Weinberg) >10^-6^, resulting in 655,658 autosomal SNPs for analysis. IMPUTE v0.4.2 based on HapMap2 was used for imputation. In TwinsUK, genotyping was performed on the Illumina arrays HumanHap300, HumanHap610Q, 1M-Duo and 1.2MDuo 1M. Quality control included removal of samples with a call rate <98%, heterozygosity across all SNPs ≥ 2 SD from the mean; non-European ancestry as judged by principal component analysis comparison with HapMap3 populations, and pairwise identity-by-descent probabilities indicative of sample identity errors. SNPs were excluded if *P*(Hardy-Weinberg) <10^-6^, MAF <1%, call rate <97% (for SNPs with MAF ≥5%) or <99% (for 1% ≤ MAF < 5%). Data sets were aligned to HapMap2 or HapMap3 forward-strand alleles. Imputation was performed with IMPUTE2 in reference to HapMap2, release 22 (combined CEU+YRI+ASN panels). 534,665 autosomal SNPs were included in the analysis. Association testing was carried out using linear regression models for log-metabolite levels. In KORA, PLINK v1.06 and SNPTEST (<https://mathgen.stats.ox.ac.uk/genetics_software/snptest/snptest.html>) were used with adjustment for age and sex. Family structure in TwinsUK was adjusted for using variance components applied to a score test in MERLIN (<http://csg.sph.umich.edu//abecasis/Merlin/index.html>). Bonferroni correction for multiple testing was applied.

***CHARGE.*** Briefly (details are available here [7]), genotyping in ARIC, CARDIA, and MESA was done on the Affymetrix 6.0, in CHS on the Illumina 370, and in InCHIANTI on the Illumina 550. Quality control excluded samples with call rates <95% (ARIC, CARDIA, and MESA) or <97% (CHS, InCHIANTI), *P*(Hardy-Weinberg) <10^-5^ (CHS, ARIC) or *P*(Hardy-Weinberg) <10^-4^ (CARDIA, InCHIANTI), and MAF <1%. Separate GWAS for fasting fatty acid levels were done in each study in linear regression models adjusted for age, sex, and site of recruitment where appropriate. Possible population stratification was corrected for in CARDIA, CHS, and MESA by including the 10 first principal components as covariates. GWAS results were combined in inverse variance-weighted meta-analysis via METAL. Results from Wu et al. [7] were downloaded from http://www.chargeconsortium.com/main/results.

***Finnish cohorts.*** Genotyping was carried out on the HumanHap CNV 370k array (NFBC1966), the Illumina HumanHap 610k array (DILGOM, GenMets), and a custom-generated HumanHap 670k array that largely overlapped with the HumanHap 610k array but had additional copy-number probes (YF, HBCS). Quality control involved removal of markers and samples with call rates <95%, and removal of individuals with excessive genome-wide heterozygosity, or discrepancies between reported and genotyped sex, or close relatedness. Imputation to the 1000 Genomes (March 2010 release) and HapMap3 (release 2) reference panels was done in IMPUTE. Accuracy of imputation was ascertained by comparing 316 markers to directly genotyped Cardiometabochip SNPs in the DILGOM cohort. For association testing with fasting serum metabolites, data from individuals using lipid-lowering medication or pregnant individuals were removed, and outlying metabolite values (>4 SD from the mean) were discarded. Age-, sex- and first 10 genetic principal components-adjusted metabolite values from each study normalized to SD-unit with a mean of zero were correlated against genotypes in SNPTEST. Results were combined in fixed-effects inverse variance-weighted meta-analysis using META (<https://mathgen.stats.ox.ac.uk/genetics_software/meta/meta.html>).

**5) Metabolomics profiling in ULSAM, PIVUS and TwinGene**

Metabolomics profiling by ultra-performance liquid chromatography/tandem mass spectrometry (UPLC/MS-MS) was performed in venous plasma and serum samples at the Proteomic and Metabolomics Facility at Colorado State University (Fort Collins, CO, USA) and has been described in detail elsewhere [11, 15, 16].

***Sample processing.*** Plasma samples were stored at -80 °C until analysis. Protein precipitation involved the dilution of 100 μl of thawed plasma samples with 400 μl of methanol, overnight storage at –20 °C and centrifugation for 30 min at 3,800 g and 4 °C. The supernatant was distributed across three 96-well plates that were sealed with heat-seal foil and kept at –20 °C until analysis. Plates were analyzed in random batches of two and injections randomized by plate order. Samples were analyzed in duplicates. Prior to analyzing each pair of plates, instrument maintenance (cone cleaning, mass calibration, and detector gain calibration) was performed and followed by injection of a quality control standard. Two conditioning and three quality control injections for quality ascertainment were carried out as 1 μl 20% methanol solutions with 2 μg/ml each of terfenadine, caffeine, sulfadimethoxime, and reserpine. We assessed standards for retention time (±0.05 min), mass accuracy (<3 ppm) and signal intensity (<25% of the relative SD). A Waters Acquity UPLC platform was used to analyze protein-precipitated samples in 1 μl injections. For separation, we used a Waters Acquity UPLC C8 column (1.8 μM, 1 mm x 100 mm) set up with a gradient from solvent A (95% water, 5% methanol, 0.1% formic acid) to solvent B (95% methanol, 5% water, 0.1% formic acid). Injections were made in 100% A (held for 6 sec), ramped to 40% B (54 sec), to 70% B (2 min), and to 100% B (8 min). The mobile phase was kept at 100% B for 6 min, reset to starting conditions over 6 sec, and allowed to equilibrate for 5.9 min. The constant flow rate was 140 μl/min. The separation column and sample were kept at 50 °C and 10 °C, respectively. The effluent was infused into an electrospray source-fitted Waters Xevo G2-TOF-MS operated in positive ion mode at a scanning m/z range of 50–1,200 at a rate of 5 Hz. Scans were alternatively performed at collision energies 6 V and 15-30 V. Sodium formate solution was injected for calibration prior to sample testing (mass accuracy 1 ppm). The capillary voltage was 2,200 V, the source and desolvation temperatures were 150 °C and 350 °C, respectively, with a nitrogen desolvation gas flow rate of 800 l/h. The quadrupole collision energy was 6 V. Waters’ DataBridge software was used to convert raw data to .cdf files.

***Data processing.*** Metabolic features were detected, aligned, grouped, and imputed using XCMS (<https://metlin.scripps.edu/xcms/> [17]) in R. Simulations of 20-40 randomly selected samples with duplicate or triplicate injections informed parameter selection over a reasonable range around the values suggested by the software developers. We used the following functions and parameters chosen to maximize intra-replicate correlation (other than the default settings): for peak detection, we used xcmsSet( ) - *method = “centWave”, ppm = 25, peakwidth = c(2:15), snthresh = 8, mzCenterFun = “wMean”, integrate = 2, mzdiff = 0.05, prefilter = c(1,5)*; for peak alignment, we used rector( ) – *method = “obiwarp”, plottype = “deviation”*; for peak grouping, we used group( ) *– bw = 2, minfrac = 0.05, max = 100, mzwid = 0.01*; and for peak filling, we used fillPeaks-chrom( ). The chosen configurations were assessed in plots generated by the peak detection and grouping algorithms. Following natural log-transformation, features were normalized in ordinary least squares linear regression models fitted for associations between feature intensity and covariates of unwanted variability (retention time correction, analysis date, sample collection, and plate effects). Subsequent analyses were based on the resultant residuals. Quality control involved manual search for samples with abnormal feature intensities and/or low Spearman correlation coefficients between duplicates and/or retention times <35 sec.

***Metabolite annotation.*** Metabolites are characterized by a set of spectral peaks with similar retention time but different mass depending on the fragmentation pattern of the parent compound. We combined strongly correlated features with shared retention times to construct representative fragmentation spectra for annotation according to the four-level accuracy approach suggested by the Metabolomics Standards Initiative [18]. Briefly, level 1 indicates an unequivocal identification based on matching by mass, fragmentation pattern, and retention time to an in-house standard, whilst Level 4 designates unknown metabolites, which could not be matched in public databases according to spectral and/or mass and/or retention time similarities with named metabolites (Level 2) or chemical classes (Level 3). Exhaustive one-by-one feature evaluation yielded 192 annotated metabolites on which the current study is based. Further details about the annotation steps are found in [15]. Full metabolomics data are available in the MetaboLights archive (study identifiers MTBLS90 for PIVUS, MTBLS124 for ULSAM, MTBLS93 for TwinGene; <http://www.ebi.ac.uk/metabolights/>).

**6) Metabolomics profiling in the replication cohorts KORA/TwinsUK, CHARGE and the Finnish consortium**

In KORA/TwinsUK, metabolomics analysis and data processing was carried out by the commercial supplier Metabolon, Inc. (Durham, NC, USA; <http://www.metabolon.com/>). Their platform combines positive and negative ion-mode ultrahigh-performance liquid chromatography/tandem mass spectrometry (UHPLC/MS2) optimized for basic and acidic compounds, as well as gas chromatography/mass spectrometry. Sample analysis following protein precipitation in methanol included two consecutive injections of 12 min each (<12% median process variability across all compounds). Spectra were compared against a standard library based on retention time, m/z, and fragmentation pattern. Quality control included removal of features with >300 missing values and data points that differed by more the 3 SD from the mean, resulting in the quantification and identification of 276 metabolites in KORA and 258 metabolites in TwinsUK. Further details are reported elsewhere [5, 14].

Fatty acid analysis in the CHARGE cohorts involved fasting plasma phospholipid isolation by thin-layer chromatography followed by quantification of fatty acids by targeted gas chromatography. In the InCHIANTI cohort, fasting plasma fatty acids were quantified in total plasma by gas chromatography [7].

In the Finnish consortium, all five cohorts had serum samples analyzed on the same nuclear magnetic resonance set-up in a central laboratory. The platform has been described previously [8, 19] and combines three ^1^H-nuclear magnetic resonance spectral windows for lipoprotein, low-molecular-weight metabolite, and lipid species, respectively. Prior to quantitative analysis, raw data were pre-processed with automated baseline zeroing, peak alignment and correction for albumin background and validated against high-performance liquid chromatography data.

**7) Assessment of MR analysis limitations**

Genetic confounding such as population stratification, canalization and pleiotropy can invalidate MR analysis [20]. We tried to limit the influence of confounding factors as far possible, but the results from the current study and other MR studies require careful interpretation and ideally experimental validation.

Population stratification: The risk of genetic confounding through population stratification is small in our study as the MR discovery samples were ethnically homogeneous, consisting largely of middle- to advanced-aged Swedes of European decent. We furthermore adjusted models for the three most important genetic principal components in the discovery cohorts PIVUS and TwinGene. Genetic analyses in the replication studies minimized potential population stratification effects by individually adjusting for the most important genetic principal components in association tests. In sensitivity analysis, additional adjustment for the first three genetic principal components in PIVUS/TwinGene changed causal estimates only to a minimal degree.

Canalization: Compensatory mechanisms during development that counteract any adverse effects from a genetic predisposition may occur. Canalization is less likely to affect genetic traits with less severe consequences or whose adverse effects become apparent later in life. No approaches are currently available to assess or control for canalization. Regarding IR and impaired insulin secretion, whose adverse metabolic consequences are slowly progressive, become largely apparent in later life and are tolerated for long periods, the effect of canalization is presumably small for associations with plasma metabolite levels.

Trait heterogeneity: A genetic variant can affect multiple aspects of a trait. For instance, it could be hypothesized that the genetic variant near *IRS1* that is included in the IR genetic risk score exerts its effect predominantly through direct interference with glucose metabolism, while the variant near *PPARG* acts mainly through transcriptional regulation. In the case of IR and impaired insulin secretion, possible trait heterogeneity in genetic associations is unlikely to affect causal inference, because a) we use a genetic risk score which included variants implicated in different molecular pathways; b) we do not distinguish between subtypes of IR and any differential effects are likely to be balanced across subjects in our samples; and c) no clear trait heterogeneity in IR and impaired insulin secretion has – to our knowledge – been established.

Winner’s curse: In GWAS that involve testing thousands of variants for trait associations and select the most strongly associated (lead) SNPs for regions in high linkage disequilibrium, chance correlations between SNPs and confounders can lead to overestimation of the effect [21]. If the GWAS discovery set and the MR sample do not overlap, this bias does not affect the size or power of the IV analysis test, but can bias causal estimates toward the null [20]. There is no overlap between the genetic discovery cohorts used by Scott et al. and the MR samples in our study thus minimizing the risk of overestimating causal effects. A potential bias toward more conservative or null effects cannot be excluded.

Horizontal pleiotropy: Vertical (mediated) pleiotropy describes the sequence where a genetic instrument affects multiple traits in a causal chain (e.g., genetic IR may lead to ectopic lipid accumulation in visceral organs which predisposes to cardiovascular disease). Horizontal (mosaic) pleiotropy describes the effect of a genetic variant on multiple traits acting at the same physiologic level (e.g., a variant in the *FADS1* gene may independently affect different lipid metabolites that may influence cardiovascular health independently). Whilst vertical pleiotropy does not invalidate MR assumptions, horizontal pleiotropy is problematic [22]. We tried to minimize bias in causal estimates through horizontal pleiotropy by using the validated genetic risk score reported by Scott et al. [10] and through extensive MR sensitivity analyses which did not provide evidence for pleiotropic interference. Adiposity, as assessed by BMI, deserves particular attention as it has been well described that genetically worse IR is linked to a reduced BMI [10, 23]. As reported in the genetic discovery analysis by Scott et al. [10] and reproduced in our results, genetic IR is associated with lower BMI. Scott et al. applied stringent criteria to exclude pleiotropic interference from BMI by, e.g., excluding any variants from the *FTO* locus and demonstrating a BMI-independent association with type 2 diabetes. They also found evidence that the negative associations between genetic IR and BMI appeared to be mediated by ectopic lipid accumulation in metabolically unfavorable body parts. This would imply mediated/vertical pleiotropy, in that genetic IR leads to ectopic fat accumulation (i.e. in visceral or hepatic depots rather than subcutaneous tissues), which has metabolically adverse effects but reduces BMI due to altered lipid deposition. The type of causal chain-type association does not violate MR assumptions and may indeed support its validity [24].

**8) Power calculation for MR analysis**

We implemented power calculation for MR analysis using the *mRnd* package in R [25]. For all significant findings (oleic acid and palmitoleic acid), we obtained post-hoc power estimates based on the obtained effects. However, to our knowledge, no readily available method for power estimation in MR analysis using multiple samples has been proposed. For instance, the observational risk factor-outcome associations were established in ULSAM, the causal effects and genetic-outcome associations were estimated in the respective MR cohorts (e.g., CHARGE), and the genetic-risk factor associations derived from either the Scott et al. publication or the MAGIC consortium. For post-hoc power analysis, we based estimates on the number of subjects in the respective MR cohort, an alpha error level of 0.05 and r^2^ = 0.02 for the proportion of risk factor variance explained by the genetic instrument. The post-hoc power estimates to detect causal effects based on the observed values for palmitoleic acid were 0.55 (PIVUS/TwinGene), 0.96 (KORA/TwinsUK), and 0.76 (CHARGE). The respective estimates for oleic acid were 0.13, 0.99, and 0.99. A priori calculations of MR sample size requirements based on alpha = 0.05, power = 0.8, r^2^ = 0.02, and a beta for the observational association between risk factor and outcome between 0.05 - 0.3 SD were: n_required_ = 8,977 – 9,862 (true causal effect of risk factor on outcome 0.2 SD); n_required_ = 16,162 - 17,345 (causal effect 0.15 SD); n_required_ = 37,082 – 38,852 (causal effect 0.1 SD). As emphasized in the main text, our analysis – particularly the MR discovery set (n = 2,613) – may have suffered a lack of power to detect true positives.

**9) MR results and demographics in KORA F4 non-diabetic persons**

Replication of MR results for all cases of significant (p < 0.05) causal effects of genetic IR on metabolite levels in any of studies was attempted in non-diabetic persons in KORA S4 (see Methods section of the main text for details of MR analysis). Regression coefficients were based on log_2_(metabolite level) transformed to SD-unit, adjusted for age, sex, and study-specific covariates. Prevalent cases of diabetes were excluded based on the presence of any of the following: fasting plasma glucose ≥7.0 mmol/l, 2-hour OGTT glucose ≥11.1 mmol/l, self- or healthcare professional-reported diagnosis of diabetes, or current anti-diabetic medication. Baseline characteristics of 1,432 non-diabetic persons (mean ± SD) were BMI 27.63 ± 4.53 kg/m^2^, total cholesterol 224 ± 39 mg/dl, HDL-cholesterol 58 ± 15 mg/dl, LDL-cholesterol 142 ± 35 mg/dl, triglycerides 126 ± 87 mg/dl, waist circumference 94.03 ± 13.67 cm, systolic/diastolic blood pressure 123.53 ± 18.35 / 76.11 ± 9.84 mmHg, and C-reactive protein level 2.20 ± 3.49 mg/l.

| **MR analysis results in 1,432 non-diabetic persons in KORA S4** | | | |
| --- | --- | --- | --- |
| **Metabolite** | **Causal estimate** | **SE** | ***P-value*** |
| Palmitoleic acid | -1.140 | 0.506 | 0.024 |
| Oleic acid | -0.820 | 0.472 | 0.082 |
| L-tyrosine | -0.007 | 0.424 | 0.987 |
| Hippuric acid | 0.133 | 0.430 | 0.757 |

**10) Gene expression look-up for *scd1***

We studied gene expression data for stearoyl-CoA desaturase 1 (*SCD1*) and its rodent equivalent *scd1* in the EMBL-EBI Expression Atlas v3.0 (<http://www.ebi.ac.uk/gxa/home>). Among all 252 uploaded experiments that reported 5%-FDR-significant different expression between conditions, we extracted all experimental manipulations that had any bearing on IR (defined as manipulations reported as changing IR status in previous research).

| **Organism** | **Experiment** | **Comparison** | **Expected effect on IR** | ***scd1* expression** | **log_2_-fold change** | **FDR-adjusted p-value** | **Array** | **Tissue** | **PMID** |
| --- | --- | --- | --- | --- | --- | --- | --- | --- | --- |
| Mus musculus | E-GEOD-5475 | PPARa knockout vs. WT on diet with PPARa-ligand WY14,643 | worse | down | -4.1 | 9.63E-08 | Affymetrix GeneChip Mouse Genome 430A 2.0 | small intestine | 17426115 |
| Mus musculus | E-MEXP-893 | high fat vs. normal diet | worse | down | -3.8 | 1.67E-06 | Affymetrix GeneChip Mouse Expression Array MOE430A and MOE430B | liver | 17618414 |
| Mus musculus | E-GEOD-5475 | diet with PPARa-ligand WY14,643 vs. control diet in WT | better | up | 3.7 | 1.29E-10 | Affymetrix GeneChip Mouse Genome 430A 2.0 | small intestine | 17426115 |
| Mus musculus | E-GEOD-2192 | PPARy2 overexpression vs. empty vector | better | up | 3.3 | 9.75E-07 | Affymetrix GeneChip Murine Genome U74Av2 | NIH3T3 adipocytes | 16106032 |
| Mus musculus | E-GEOD-38138 | streptozotocin-induced diabetes vs. control | worse | down | -3 | 0.006 | Affymetrix GeneChip Mouse Genome 430 2.0 | liver | 19413312 |
| Rattus norvegicus | E-GEOD-2470 | diabetes mellitus vs. normal (both treated with sodium tungstate | worse | down | -1.2 | 0.007 | Affymetrix GeneChip Rat Expression Array RAE230A | pancreas | 19715561 |
| Mus musculus | E-GEOD-20636 | diabetes mellitus vs. normal (8mo.) | worse | down | -1.1 | 0.004 | Affymetrix GeneChip Mouse Genome 430 2.0 | kidney | 21606656 |
| Mus musculus | E-GEOD-20636 | diabetes mellitus vs. normal (4mo.) | worse | down | -1.1 | 0.007949256 | A-AFFY-45 - Affymetrix GeneChip Mouse Genome 430 2.0 | kidney | 21606656 |

**11) Comparison between whole plasma metabolomics and serum cholesteryl ester fatty acid analysis in ULSAM and PIVUS**

To provide an additional level of confidence in our in-house publicly available analysis pipeline, we reasoned that plasma fatty acid measurement by untargeted metabolomics should show adequate overlap with the targeted and validated serum cholesteryl ester (CE) fatty acid composition that was measured in a subset of persons in ULSAM/PIVUS. As previous studies have shown a strong overlap between CE and free phospholipid composition [26], we used Bland-Altman-type comparisons between both assays, which demonstrated good agreement for oleic and palmitoleic acid. Bland-Altman plots illustrate the SD unit-standardized comparisons between metabolomics and serum cholesteryl ester analysis in ULSAM (n = 1,138) and PIVUS (n = 970). For the latter analysis, frozen serum sample (-70 °C) were unthawed for chloroform extraction followed by placement on thin LC plates to separate lipids. Trans-methylated lipid esters were extracted, dissolved in hexane and analyzed by gas-liquid chromatography by a Hewlett Packard GLC system (components 5890, 7671A and 3392A). Fatty acids were identified by retention time comparisons to reference standards. The figures below show scatter and Bland-Altman plots separately in ULSAM and PIVUS for oleic and palmitoleic acid and indicate directionally consistent, reasonable agreements between both analysis platforms.

**References**

1. Magnusson PK, Almqvist C, Rahman I, Ganna A, Viktorin A, Walum H, et al. The Swedish Twin Registry: establishment of a biobank and other recent developments. Twin Res Hum Genet. 2013;16(1):317-329.

2. Ganna A, Reilly M, de Faire U, Pedersen N, Magnusson P, Ingelsson E. Risk prediction measures for case-cohort and nested case-control designs: an application to cardiovascular disease. Am J Epidemiol. 2012;175(7):715-724.

3. Hedstrand H. A study of middle-aged men with particular reference to risk factors for cardiovascular disease. Ups J Med Sci Suppl. 1975;19:1-61.

4. Lind L, Fors N, Hall J, Marttala K, Stenborg A. A comparison of three different methods to determine arterial compliance in the elderly: the Prospective Investigation of the Vasculature in Uppsala Seniors (PIVUS) study. J Hypertens. 2006;24(6):1075-1082.

5. Shin SY, Fauman EB, Petersen AK, Krumsiek J, Santos R, Huang J, et al. An atlas of genetic influences on human blood metabolites. 2014;46(6):543-550.

6. Illig T, Gieger C, Zhai G, Romisch-Margl W, Wang-Sattler R, Prehn C, et al. A genome-wide perspective of genetic variation in human metabolism. Nat Genet. 2010;42(2):137-141.

7. Wu JH, Lemaitre RN, Manichaikul A, Guan W, Tanaka T, Foy M, et al. Genome-wide association study identifies novel loci associated with concentrations of four plasma phospholipid fatty acids in the de novo lipogenesis pathway: results from the Cohorts for Heart and Aging Research in Genomic Epidemiology (CHARGE) consortium. Circ Cardiovasc Genet. 2013;6(2):171-183.

8. Kettunen J, Tukiainen T, Sarin AP, Ortega-Alonso A, Tikkanen E, Lyytikainen LP, et al. Genome-wide association study identifies multiple loci influencing human serum metabolite levels. Nat Genet. 2012;44(3):269-276.

9. DeFronzo RA, Tobin JD, Andres R. Glucose clamp technique: a method for quantifying insulin secretion and resistance. Am J Physiol. 1979;237(3):E214-223.

10. Scott RA, Fall T, Pasko D, Barker A, Sharp SJ, Arriola L, et al. Common genetic variants highlight the role of insulin resistance and body fat distribution in type 2 diabetes, independent of obesity. Diabetes. 2014;63(12):4378-4387.

11. Ganna A, Salihovic S, Sundstrom J, Broeckling CD, Hedman AK, Magnusson PK, et al. Large-scale metabolomic profiling identifies novel biomarkers for incident coronary heart disease. PLoS Genet. 2014;10(12):e1004801.

12. Nowak C, Sundstrom J, Gustafsson S, Giedraitis V, Lind L, Ingelsson E, et al. Protein biomarkers for insulin resistance and type 2 diabetes risk in two large community cohorts. Diabetes. 2015.

13. den Hoed M, Strawbridge RJ, Almgren P, Gustafsson S, Axelsson T, Engstrom G, et al. GWAS-identified loci for coronary heart disease are associated with intima-media thickness and plaque presence at the carotid artery bulb. Atherosclerosis. 2015;239(2):304-310.

14. Suhre K, Shin SY, Petersen AK, Mohney RP, Meredith D, Wagele B, et al. Human metabolic individuality in biomedical and pharmaceutical research. Nature. 2011;477(7362):54-60.

15. Ganna A, Fall T, Salihovic S, Lee W, Broeckling CD, Kumar J, et al. Large-scale non-targeted metabolomics profiling in three human population-based studies. Metabolomics. 2015.

16. Broeckling CD, Heuberger AL, Prenni JE. Large scale non-targeted metabolomic profiling of serum by ultra performance liquid chromatography-mass spectrometry (UPLC-MS). J Vis Exp. 2013(73):e50242.

17. Smith CA, Want EJ, O'Maille G, Abagyan R, Siuzdak G. XCMS: processing mass spectrometry data for metabolite profiling using nonlinear peak alignment, matching, and identification. Anal Chem. 2006;78(3):779-787.

18. Sumner LW, Amberg A, Barrett D, Beale MH, Beger R, Daykin CA, et al. Proposed minimum reporting standards for chemical analysis Chemical Analysis Working Group (CAWG) Metabolomics Standards Initiative (MSI). Metabolomics. 2007;3(3):211-221.

19. Soininen P, Kangas AJ, Wurtz P, Tukiainen T, Tynkkynen T, Laatikainen R, et al. High-throughput serum NMR metabonomics for cost-effective holistic studies on systemic metabolism. Analyst. 2009;134(9):1781-1785.

20. Haycock PC, Burgess S, Wade KH, Bowden J, Relton C, Davey Smith G. Best (but oft-forgotten) practices: the design, analysis, and interpretation of Mendelian randomization studies. 2016.

21. Goring HH, Terwilliger JD, Blangero J. Large upward bias in estimation of locus-specific effects from genomewide scans. Am J Hum Genet. 2001;69(6):1357-1369.

22. Paaby AB, Rockman MV. The many faces of pleiotropy. Trends Genet. 2013;29(2):66-73.

23. Yaghootkar H, Lamina C, Scott RA, Dastani Z, Hivert MF, Warren LL, et al. Mendelian randomization studies do not support a causal role for reduced circulating adiponectin levels in insulin resistance and type 2 diabetes. Diabetes. 2013;62(10):3589-3598.

24. Davey Smith G. Using Mendelian randomization. 2016.

25. Brion MJ, Shakhbazov K, Visscher PM. Calculating statistical power in Mendelian randomization studies. Int J Epidemiol. 2013;42(5):1497-1501.

26. Ma J, Folsom AR, Shahar E, Eckfeldt JH. Plasma fatty acid composition as an indicator of habitual dietary fat intake in middle-aged adults. The Atherosclerosis Risk in Communities (ARIC) Study Investigators. Am J Clin Nutr. 1995;62(3):564-571.
