# Supplementary figures and images for: Effect of Insulin Resistance on Monounsaturated Fatty Acid Levels: A Multi-cohort Non-targeted Metabolomics and Mendelian Randomization Study
Source: PLoS Genet. 2016 Oct 21;12(10):e1006379. doi: 10.1371/journal.pgen.1006379 (PMC5074591; doi:10.1371/journal.pgen.1006379)

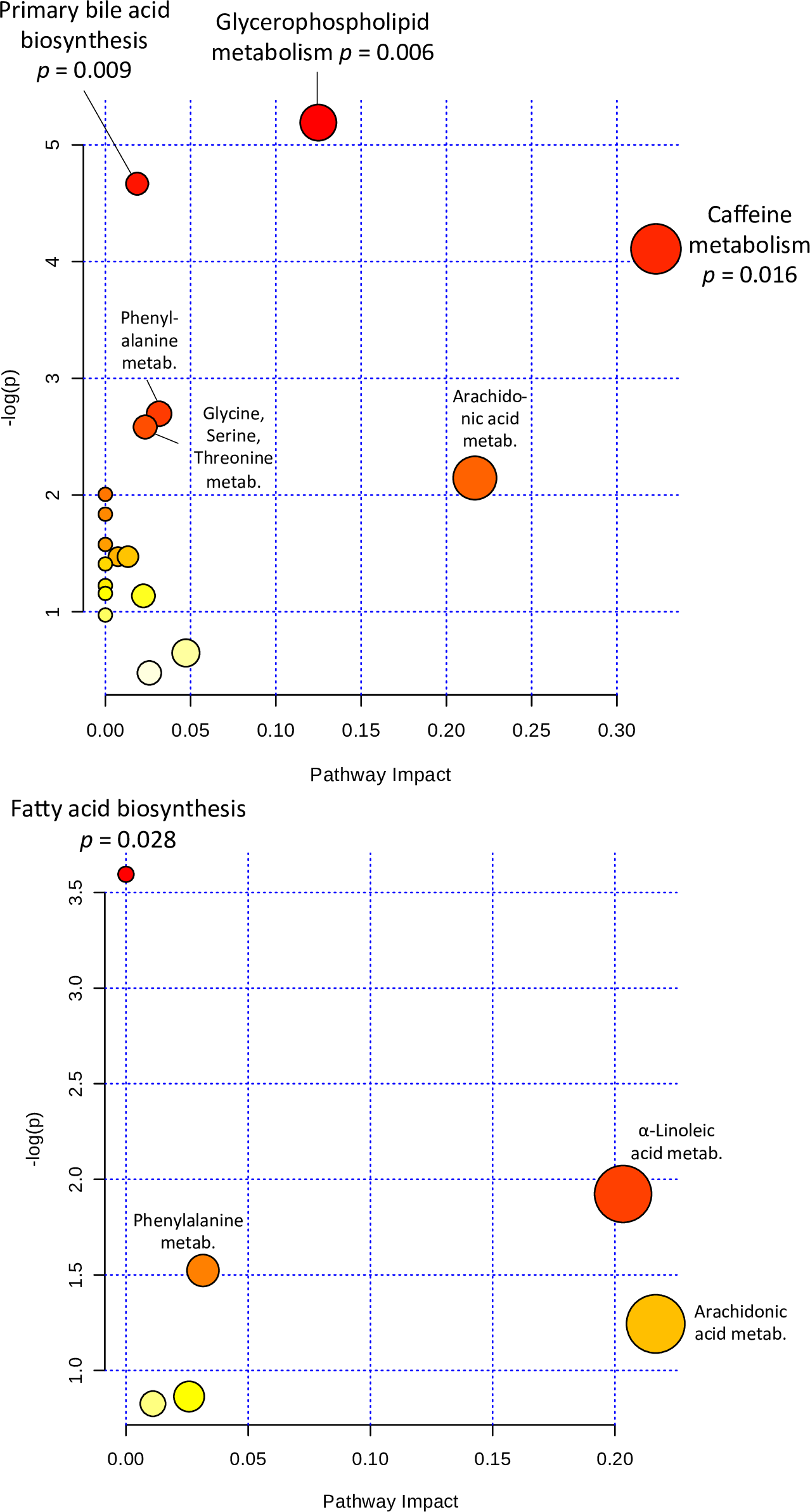

Supplement: S1 Fig — (TIF) [file pgen.1006379.s003.tif]

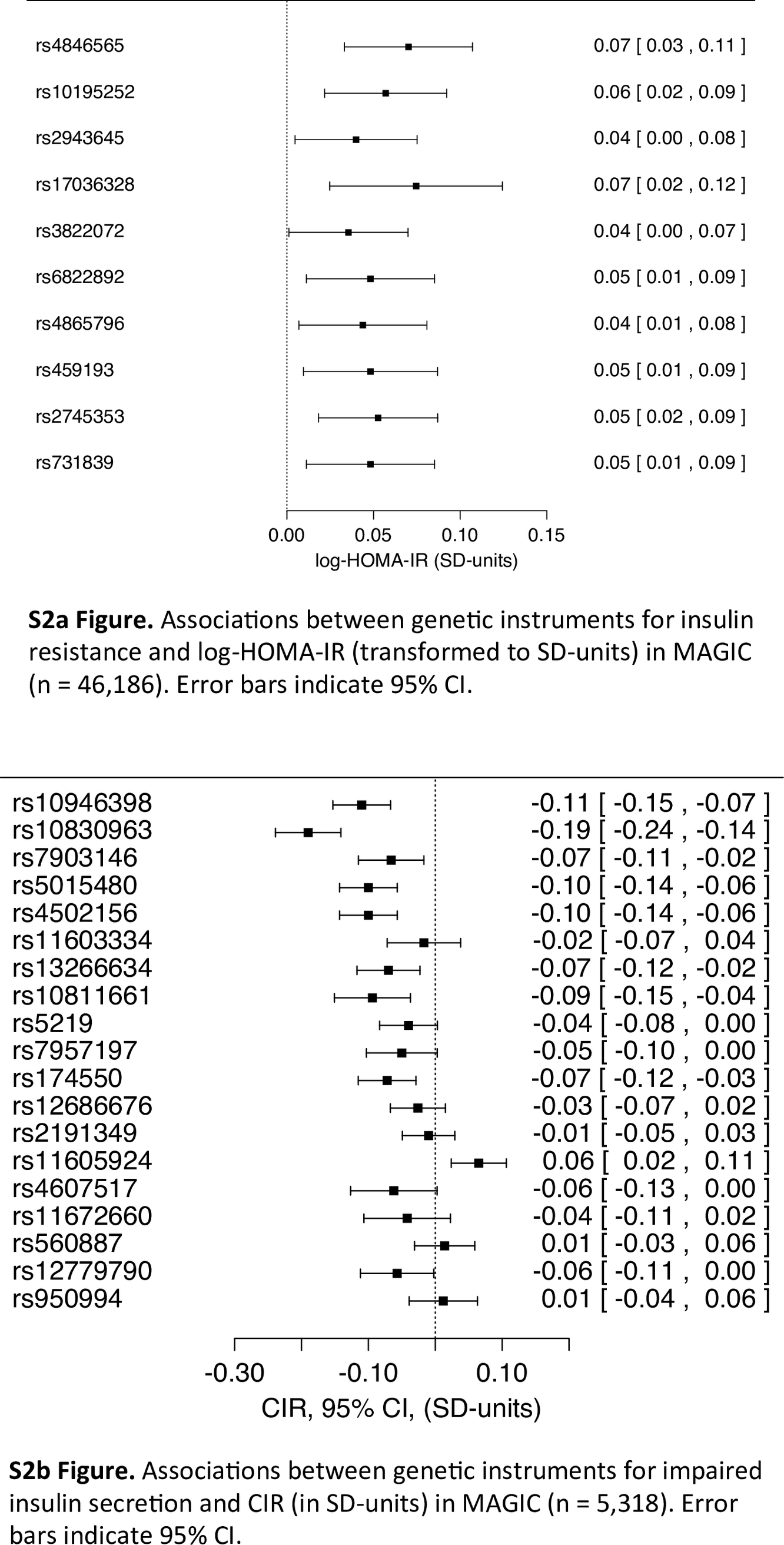

Supplement: S2 Fig — (TIF) [file pgen.1006379.s004.tif]

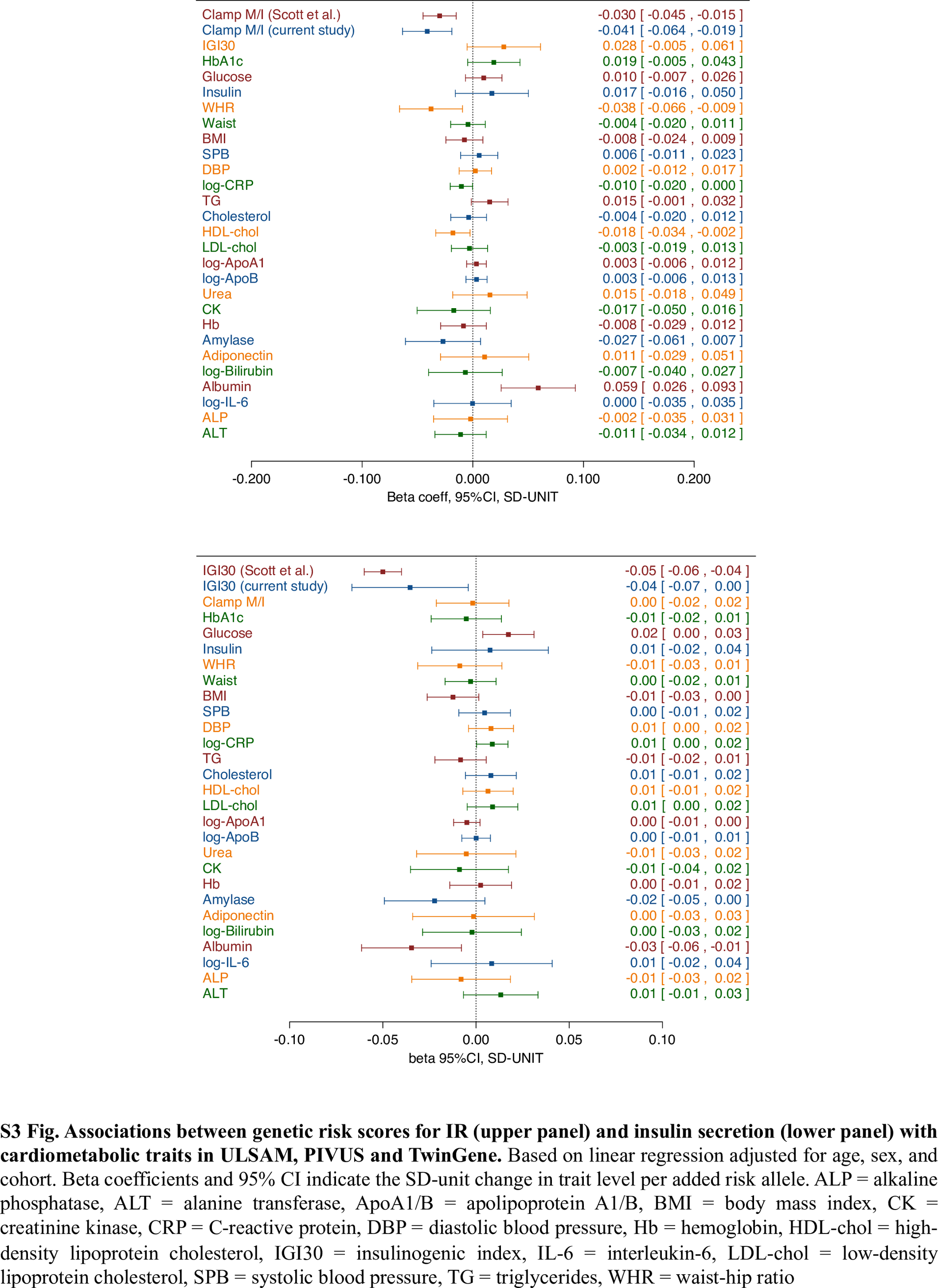

Supplement: S3 Fig — Associations between genetic risk scores for IR (upper panel) and insulin secretion (lower panel) with cardiometabolic traits in ULSAM, PIVUS and TwinGene. Based on linear regression adjusted for age, sex, and cohort. Beta coefficients and 95% CIs indicate the SD-unit change in trait level per added risk allele. ALP = alkaline phosphatase, ALT = alanine amino transferase, ApoA1/B = apolipoprotein A1/B, BMI = body mass index, CK = creatinine kinase, CRP = C-reactive protein, DBP = diastolic blood pressure, Hb = hemoglobin, HDL-chol = high-density lipoprotein cholesterol, IGI30 = insulinogenic index, IL-6 = interleukin-6, LDL-chol = low-density lipoprotein cholesterol, SBP = systolic blood pressure, TG = triglycerides, WHR = waist-hip ratio (TIF) [file pgen.1006379.s005.tif]
