# Supplementary material for: Effect of Insulin Resistance on Monounsaturated Fatty Acid Levels: A Multi-cohort Non-targeted Metabolomics and Mendelian Randomization Study
Source: PLoS Genet. 2016 Oct 21;12(10):e1006379. doi: 10.1371/journal.pgen.1006379 (PMC5074591; doi:10.1371/journal.pgen.1006379)
Supplement: S4 Fig — (PDF) [file pgen.1006379.s006.pdf]

Palmitoleic acid

PIVUS/Twingene (n=2,613)

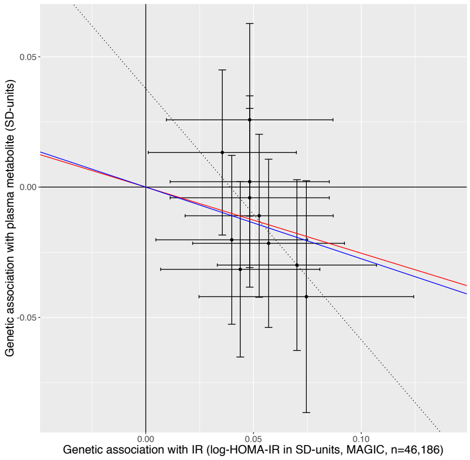

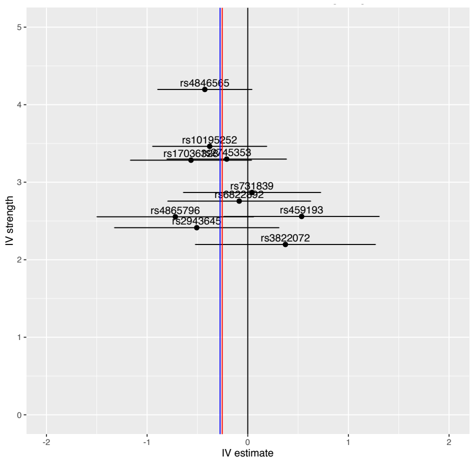

KORA/TwinsUK (n=7,776)

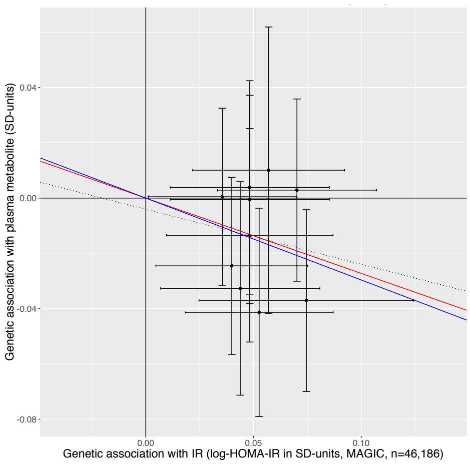

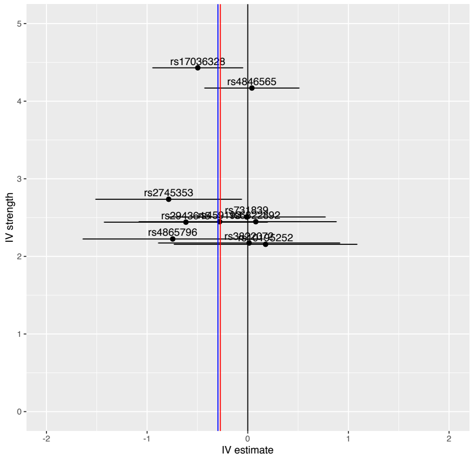

CHARGE (n=8,961)

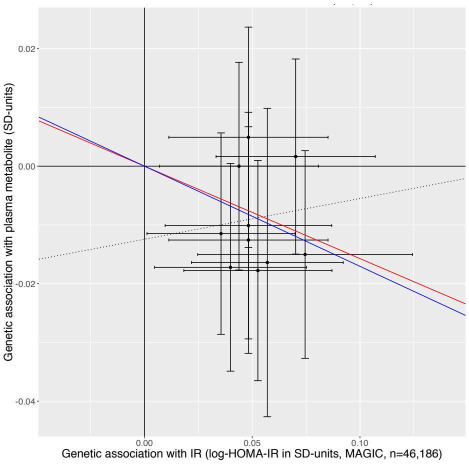

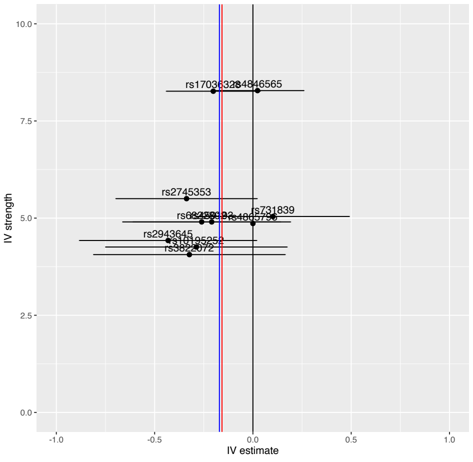

S4 Figure. Left panel: Scatter plots of genetic associations with the outcome against genetic associations with insulin resistance (log-HOMA-IR, n = 46,186) adjusted for age and sex in SD-units. Right panel: Funnel plots of instrument strength against instrumental variables estimates. Regression lines indicate instrumental variable estimates for inverse variance weighted (red), likelihood-based (blue), and Egger regression (dotted) MR analysis. Error bars indicate 95% CI.

Oleic acid

PIVUS/Twingene (n=2,613)

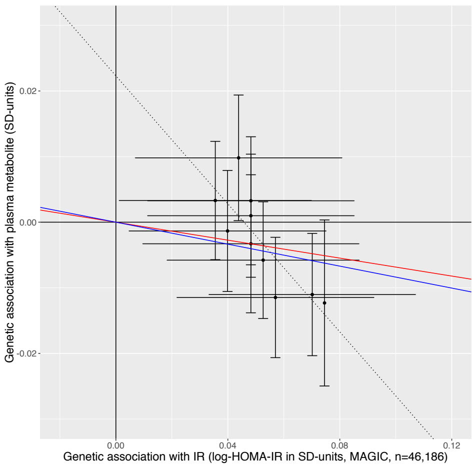

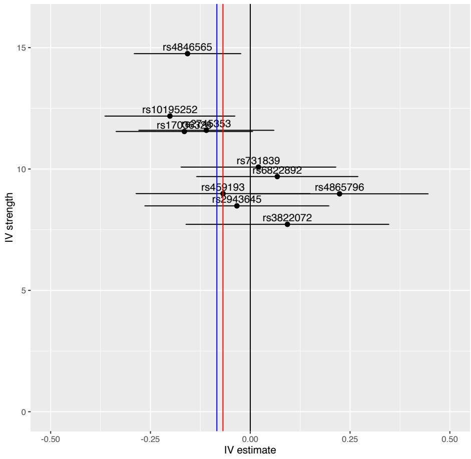

KORA/TwinsUK (n=7,768)

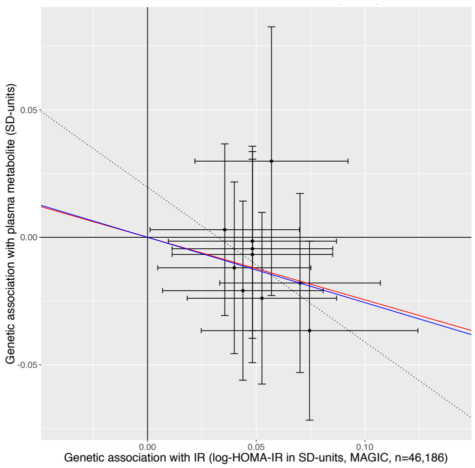

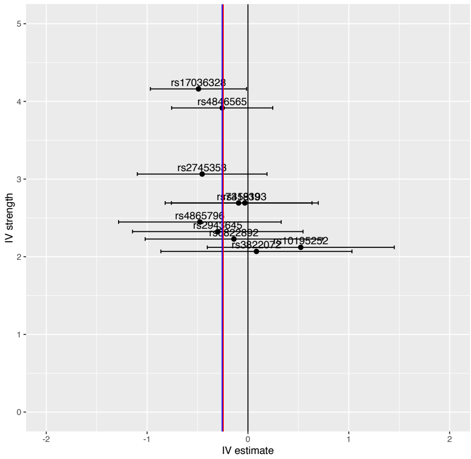

CHARGE (n=8,961)

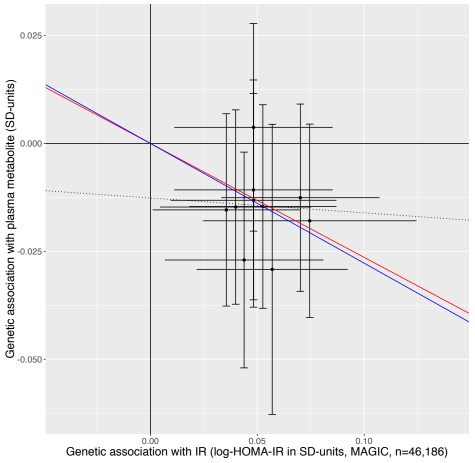

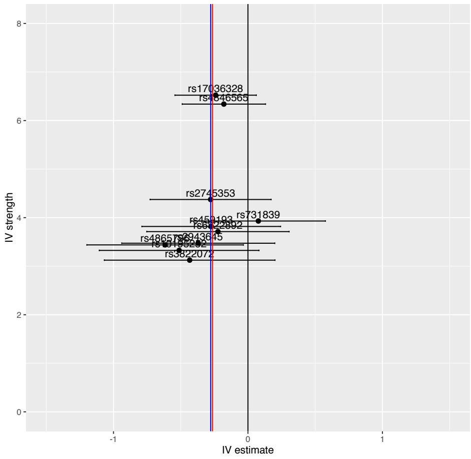

S4 Figure. Left panel: Scatter plots of genetic associations with the outcome against genetic associations with insulin resistance (log-HOMA-IR, n = 46,186) adjusted for age and sex in SD-units. Right panel: Funnel plots of instrument strength against instrumental variables estimates. Regression lines indicate instrumental variable estimates for inverse variance weighted (red), likelihood-based (blue), and Egger regression (dotted) MR analysis. Error bars indicate 95% CI.

Tyrosine

PIVUS/Twingene (n=2,613)

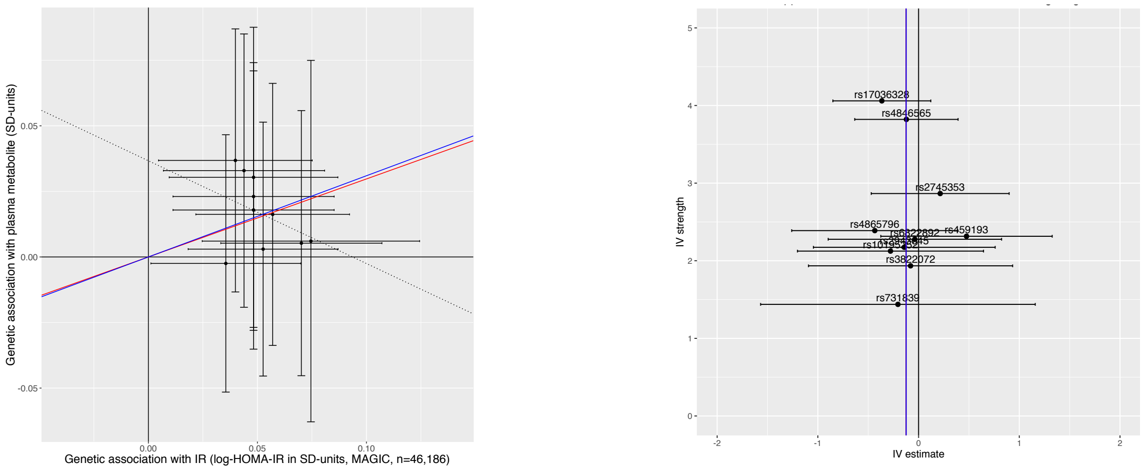

KORA/TwinsUK (n=7,807)

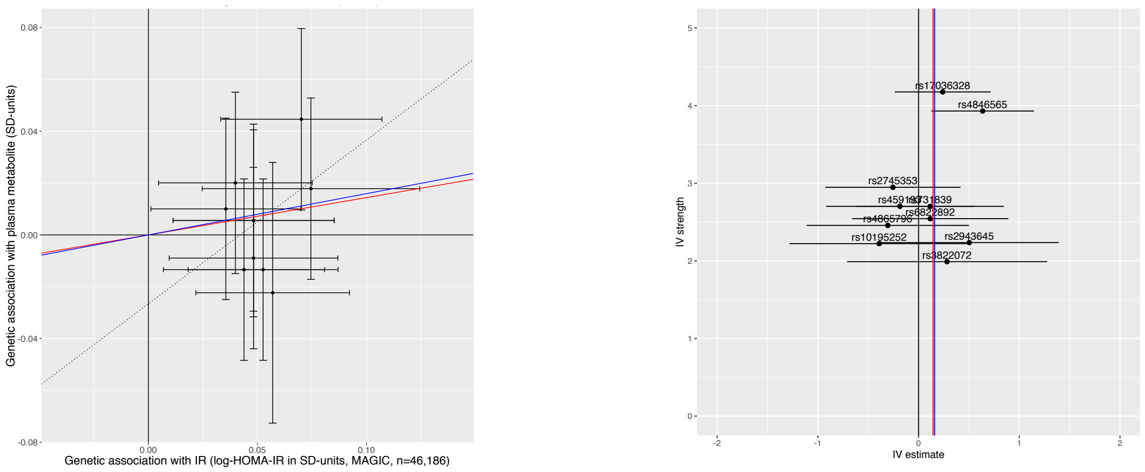

Finnish cohorts(n=8,330)

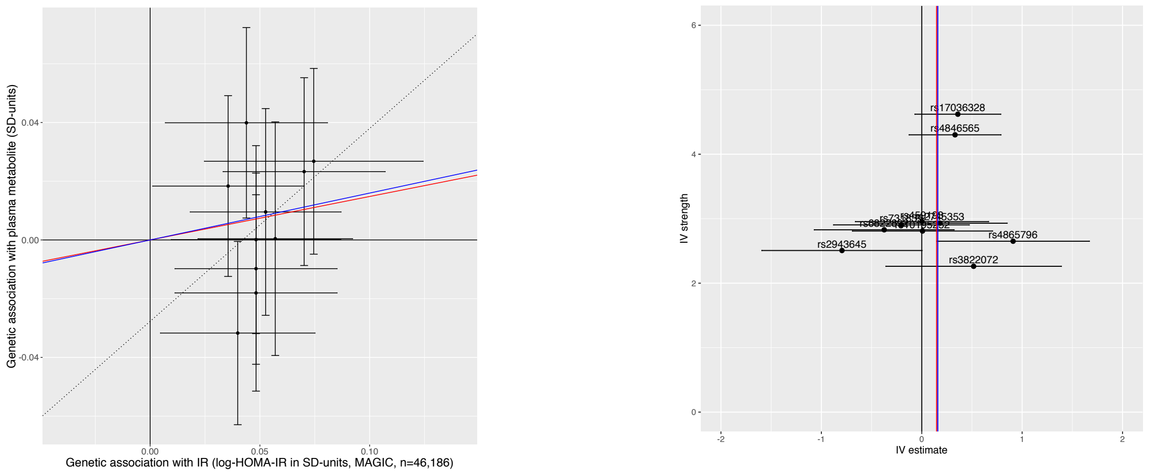

S4 Figure. Left panel: Scatter plots of genetic associations with the outcome against genetic associations with insulin resistance (log-HOMA-IR, n = 46,186) adjusted for age and sex in SD-units. Right panel: Funnel plots of instrument strength against instrumental variables estimates. Regression lines indicate instrumental variable estimates for inverse variance weighted (red), likelihood-based (blue), and Egger regression (dotted) MR analysis. Error bars indicate 95% CI.

Hippuric acid

PIVUS/Twingene (n=2,613)

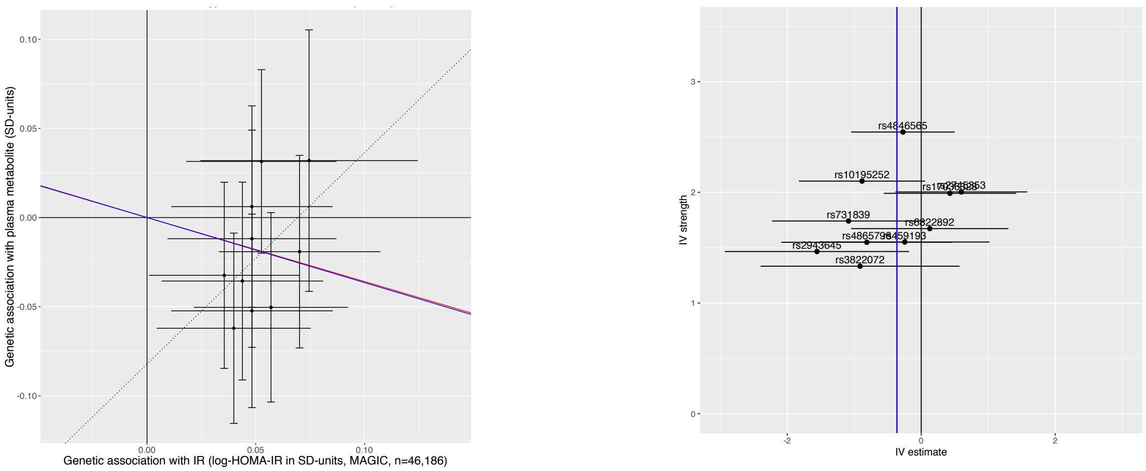

KORA/TwinsUK (n=7,806)

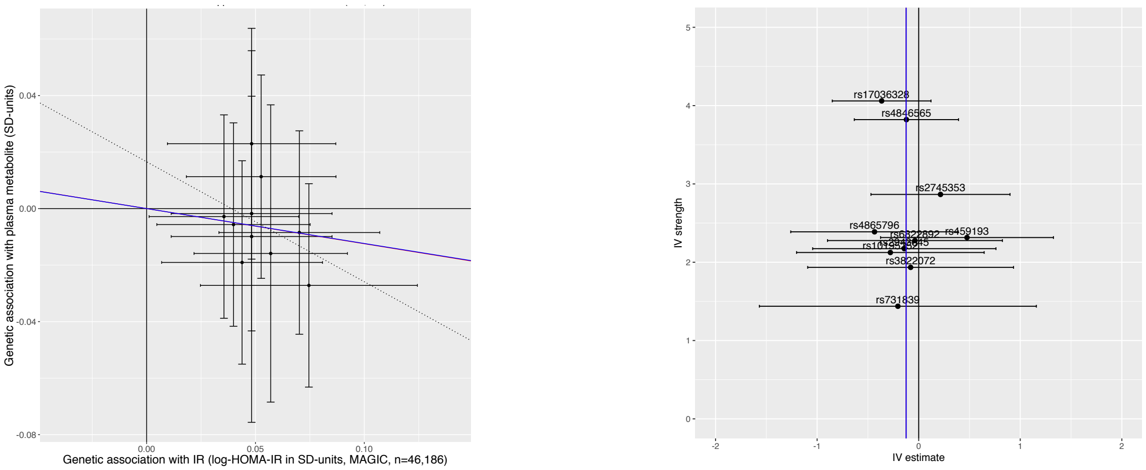

S4 Figure. Left panel: Scatter plots of genetic associations with the outcome against genetic associations with insulin resistance (log-HOMA-IR, n = 46,186) adjusted for age and sex in SD-units. Right panel: Funnel plots of instrument strength against instrumental variables estimates. Regression lines indicate instrumental variable estimates for inverse variance weighted (red), likelihood-based (blue), and Egger regression (dotted) MR analysis. Error bars indicate 95% CI.

Monoacylglycerol 18:1

PIVUS/Twingene (n=2,613)

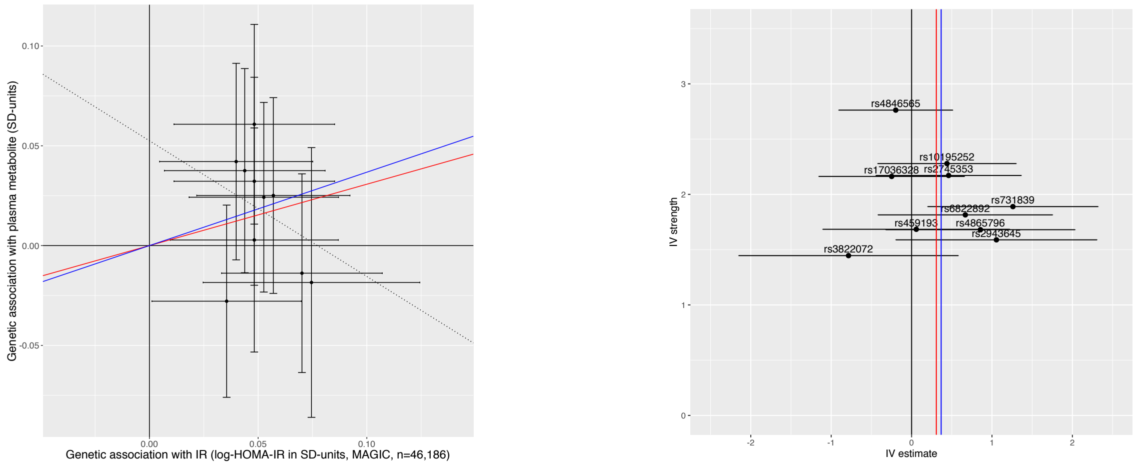

KORA/TwinsUK (n=5,717)

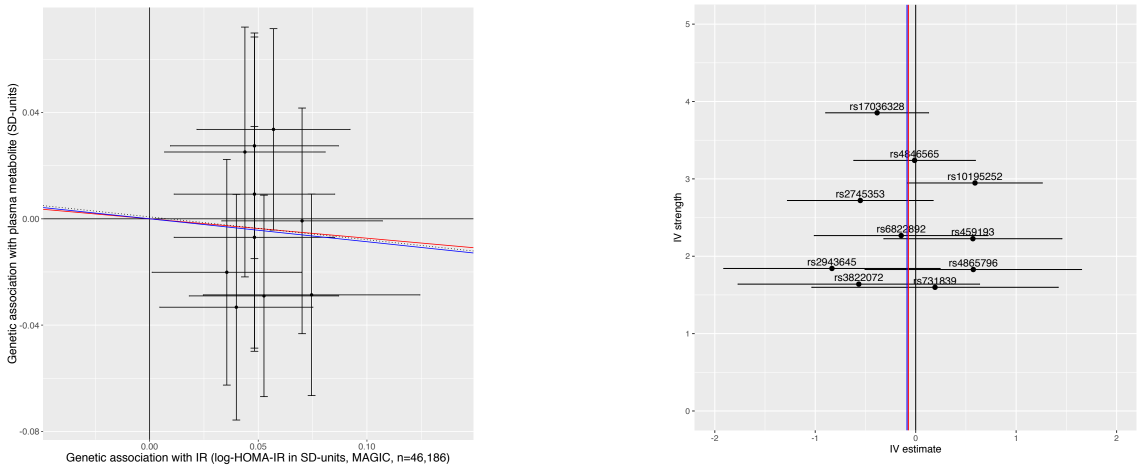

S4 Figure. Left panel: Scatter plots of genetic associations with the outcome against genetic associations with insulin resistance (log-HOMA-IR, n = 46,186) adjusted for age and sex in SD-units. Right panel: Funnel plots of instrument strength against instrumental variables estimates. Regression lines indicate instrumental variable estimates for inverse variance weighted (red), likelihood-based (blue), and Egger regression (dotted) MR analysis. Error bars indicate 95% CI.

Monoacylgycerol 18:2

PIVUS/Twingene (n=2,613)

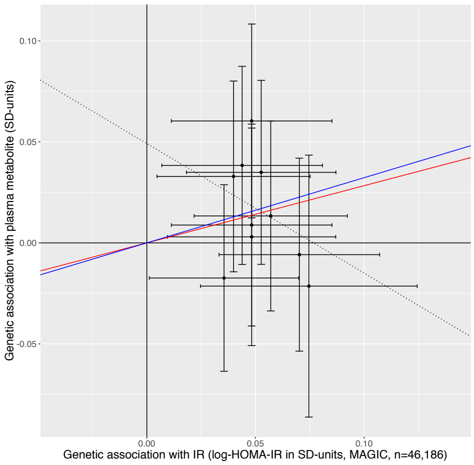

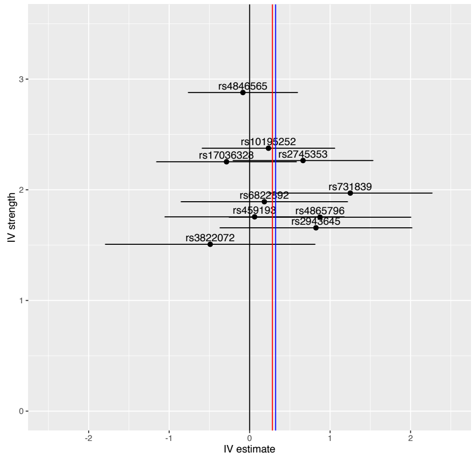

KORA/TwinsUK (n=2,797)

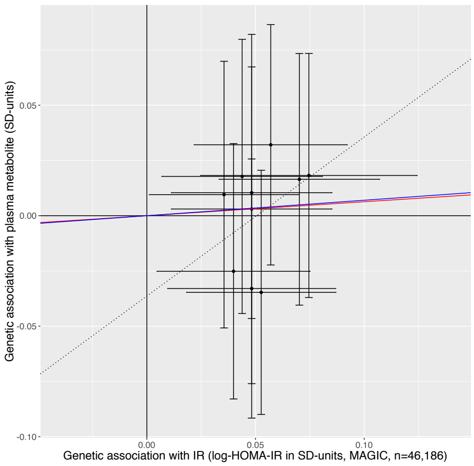

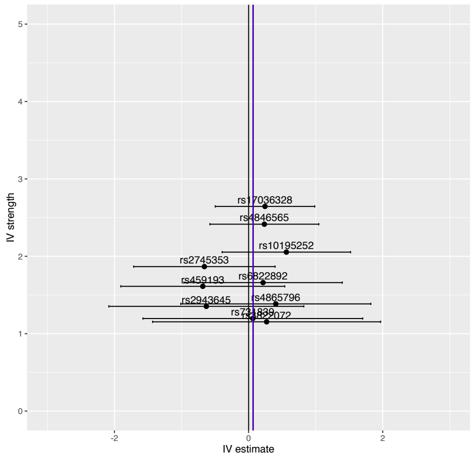

S4 Figure. <u>Left panel</u>: Scatter plots of genetic associations with the outcome against genetic associations with insulin resistance (log-HOMA-IR, n = 46,186) adjusted for age and sex in SD-units. <u>Right panel:</u> Funnel plots of instrument strength against instrumental variables estimates. Regression lines indicate instrumental variable estimates for inverse variance weighted (red), likelihood-based (blue), and Egger regression (dotted) MR analysis. Error bars indicate 95% CI.

Gamma-tocopherol

PIVUS/Twingene (n=2,613)

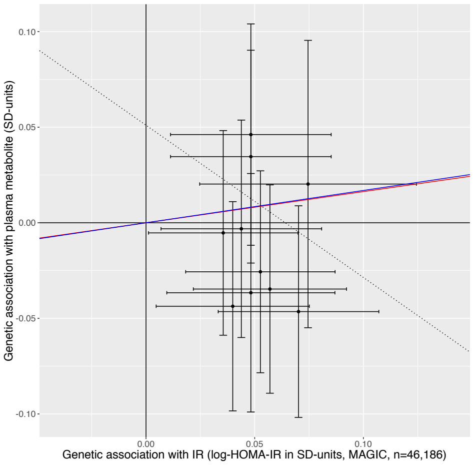

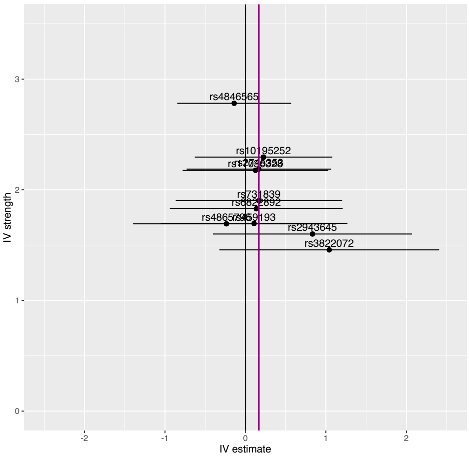

KORA/TwinsUK (n=7,203)

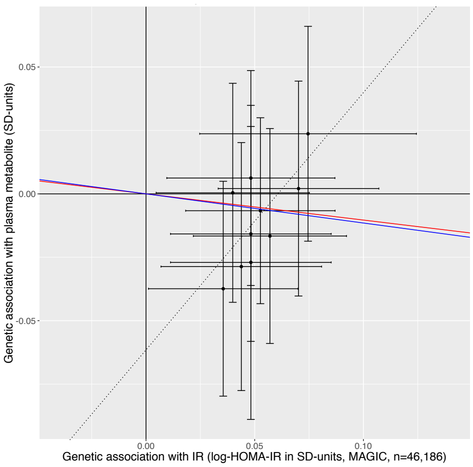

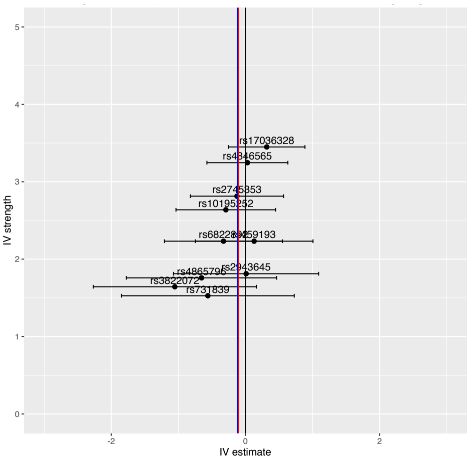

S4 Figure. <u>Left panel</u>: Scatter plots of genetic associations with the outcome against genetic associations with insulin resistance (log-HOMA-IR, n = 46,186) adjusted for age and sex in SD-units. <u>Right panel:</u> Funnel plots of instrument strength against instrumental variables estimates. Regression lines indicate instrumental variable estimates for inverse variance weighted (red), likelihood-based (blue), and Egger regression (dotted) MR analysis. Error bars indicate 95% CI.

3a,6b,7b-Trihydroxy-5b-cholanoic acid

PIVUS/Twingene (n=2,613)

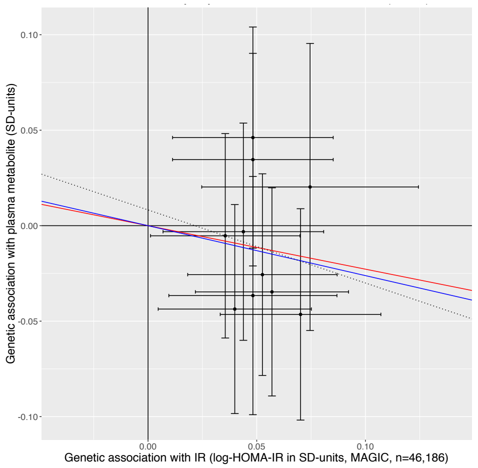

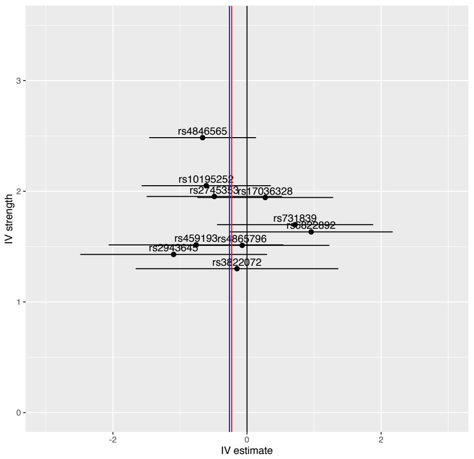

Monoacylglycerol 14:0

PIVUS/Twingene (n=2,613)

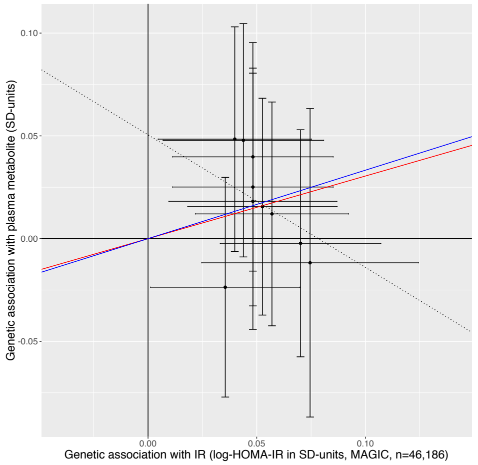

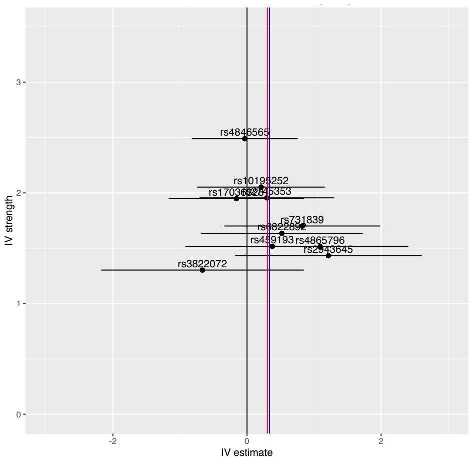

S4 Figure. Left panel: Scatter plots of genetic associations with the outcome against genetic associations with insulin resistance (log-HOMA-IR, n = 46,186) adjusted for age and sex in SD-units. Right panel: Funnel plots of instrument strength against instrumental variables estimates. Regression lines indicate instrumental variable estimates for inverse variance weighted (red), likelihood-based (blue), and Egger regression (dotted) MR analysis. Error bars indicate 95% CI.

Bilirubin

PIVUS/Twingene (n=2,613)

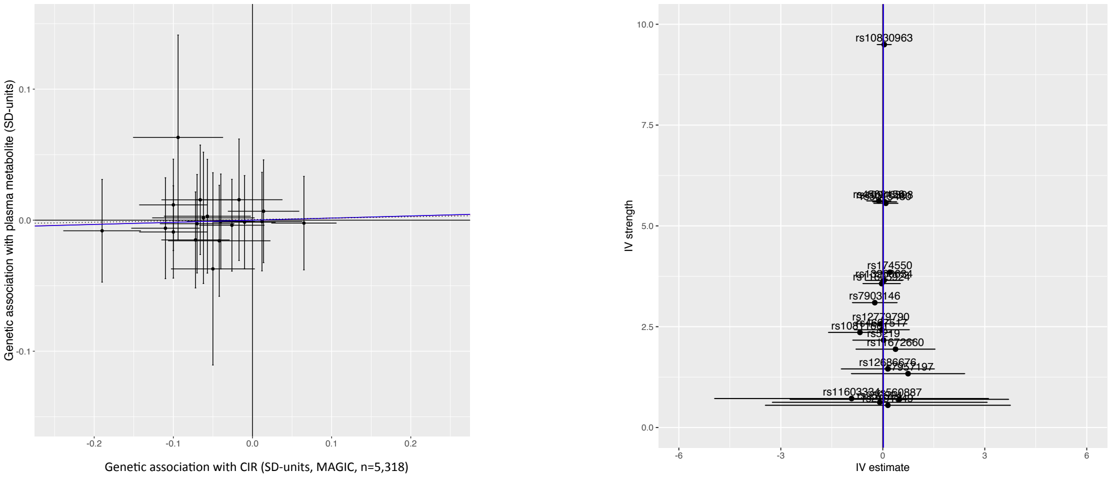

KORA/TwinsUK (n=6,812)

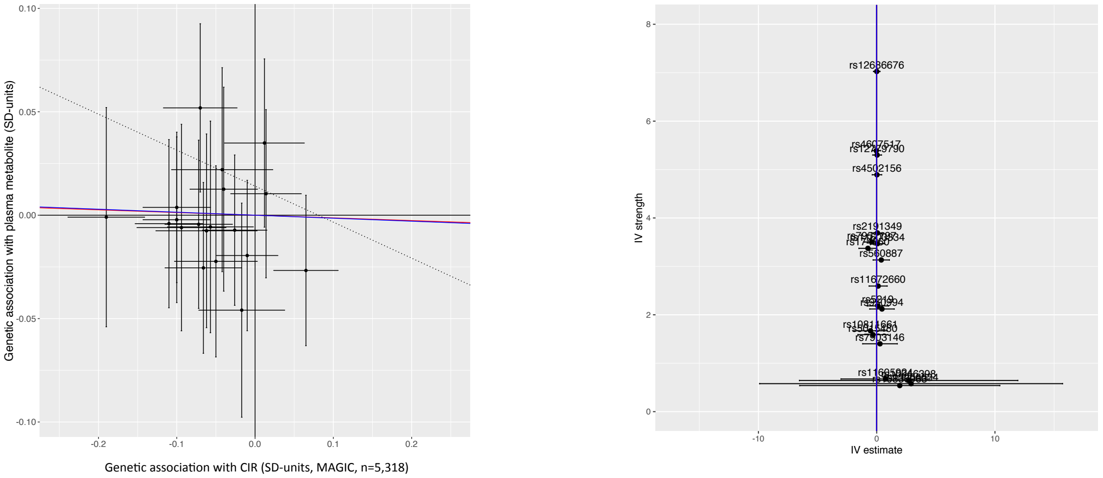

3a,6b,7b-Trihydroxy-5b-cholanoic acid

PIVUS/Twingene (n=2,613)

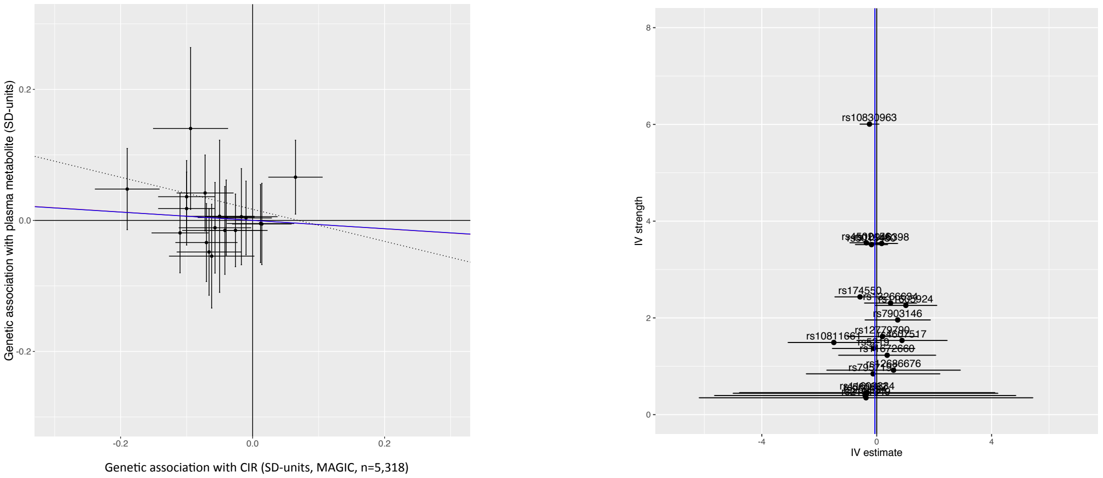

S4 Figure. Left panel: Scatter plots of genetic associations with the outcome against genetic associations with insulin secretion (CIR, n = 5,318) adjusted for age and sex in SD-units. Right panel: Funnel plots of instrument strength against instrumental variables estimates. Regression lines indicate instrumental variable estimates for inverse variance weighted (red), likelihood-based (blue), and Egger regression (dotted) MR analysis. Error bars indicate 95% CI.
