# Supplementary material for: Correction: Effect of Insulin Resistance on Monounsaturated Fatty Acid Levels: A Multi-cohort Non-targeted Metabolomics and Mendelian Randomization Study
Source: PLoS Genet. 2017 Sep 14;13(9):e1007002. doi: 10.1371/journal.pgen.1007002 (PMC5598926; doi:10.1371/journal.pgen.1007002)
Supplement: S2 Text — (DOCX) [file pgen.1007002.s001.docx]

**S2 Text.** Product ion spectra of identified metabolites in the Swedish cohorts (upper spectrum) and their corresponding standards (lower spectrum) on a UPLC-QTOFMS operated in ESI positive ion mode, as well as for tentatively identified metabolites based on comparisons to public database reference standards.

**Panel 1:** Metabolite eluting at 7.78 min represented by [2M + K]^+^ = 603.467 identified as oleic acid.

**Panel 2:** Metabolite eluting at 0.73 min represented by a fragment ion at *m/z* 166.058 identified as L-tyrosine.

**
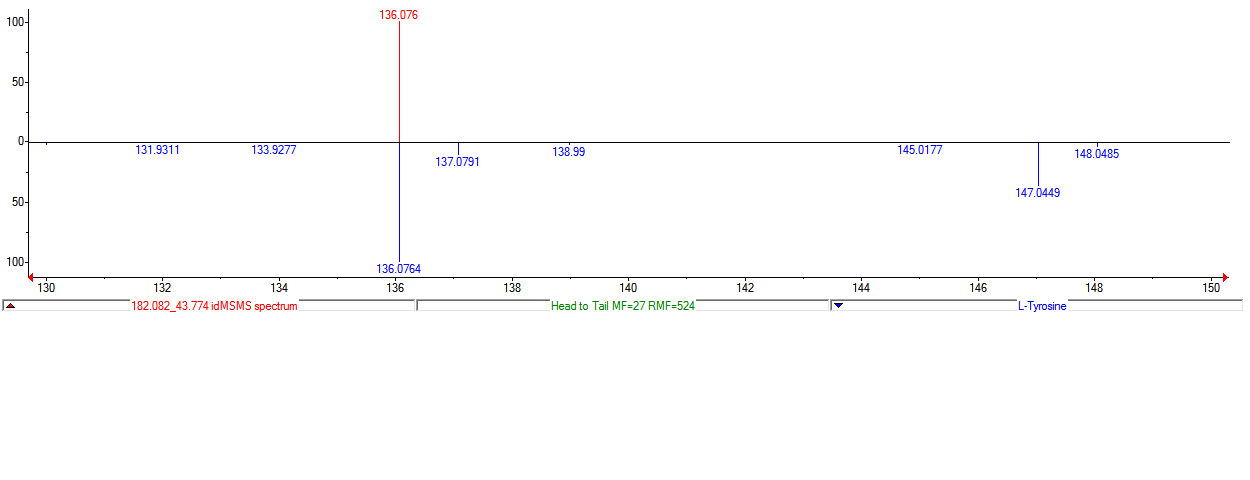
**

**Panel 3:** Metabolite eluting at 1.90 min represented by [M + H]^+^ = 202.048 identified as hippuric acid.
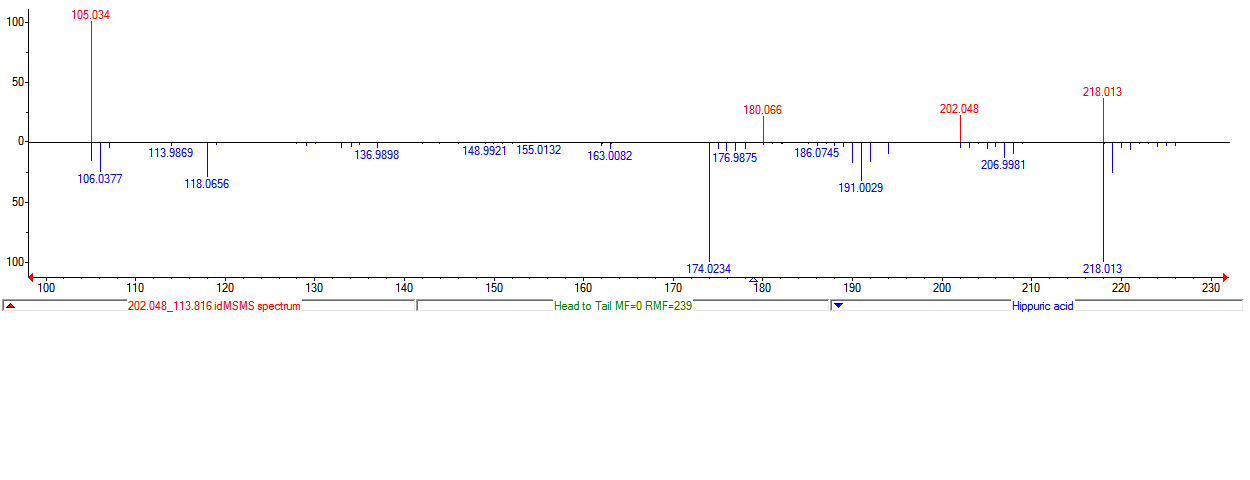


**Panel 4:** Metabolite eluting at 4.66 min represented by [M + Na]^+^ = 431.277 identified as 3a,6b,7b-trihydroxy-5b-cholanoic acid.


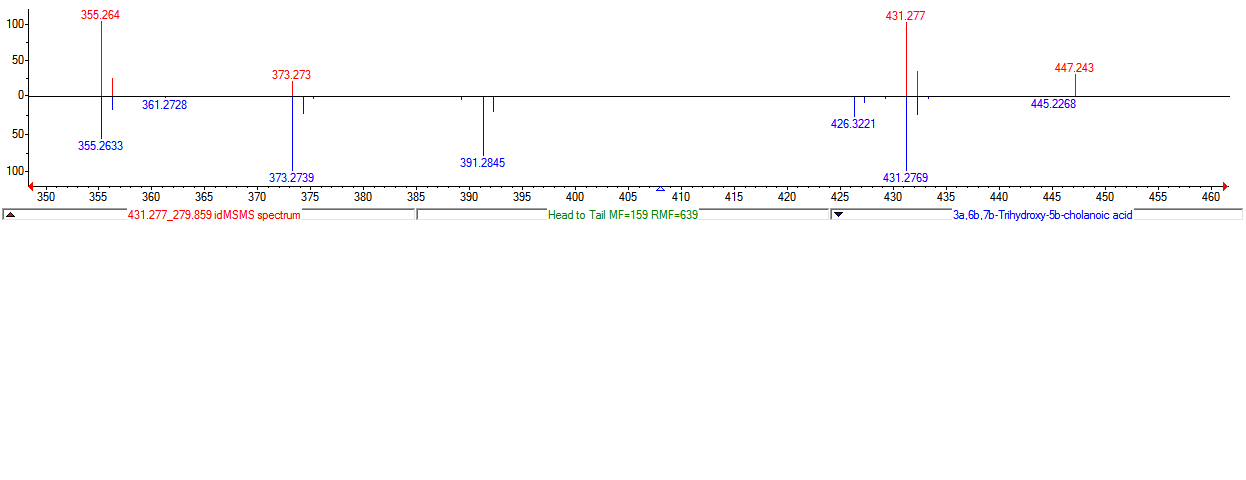


**Panel 5:** Mass spectrum of tentatively identified lysophosphatidylcholine 20:3 (C_28_H_52_NO_7_P, MW = 545.348 Da) and its fragmentation pattern using a UPLC-QTOFMS operated in ESI positive ion mode, where the protonated molecule [M + H]^+^ = 546.356 and the adduct and fragment ions at *m/z* 568.338 and *m/z* 184.074 are represented by the adduct [M + Na]^+^ as well as the loss of a phosphocholine group [C_5_H_14_NO_4_P]^+^, respectively.


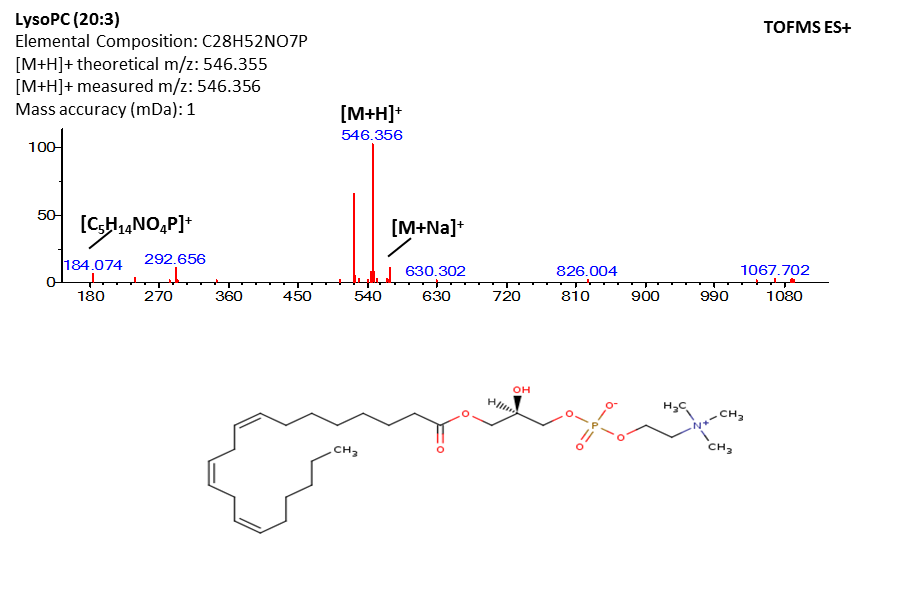


**Panel 6:** Mass spectrum of tentatively identified palmitoleic acid (C_16_H_30_O_2_, MW = 254.224 Da) and its fragmentation pattern using a UPLC-QTOFMS operated in ESI ion positive mode, where the protonated molecule [M + H]^+^ = 255.232 and the adduct and fragment ions at m/z 293.3179, m/z 277.202, m/z 237.222, m/z 219.211 are represented by adduct formation [M + K]^+^ and [M + Na]^+^ as well as the loss of water [M + H - H_2_O] and [M + H - 2H_2_O].


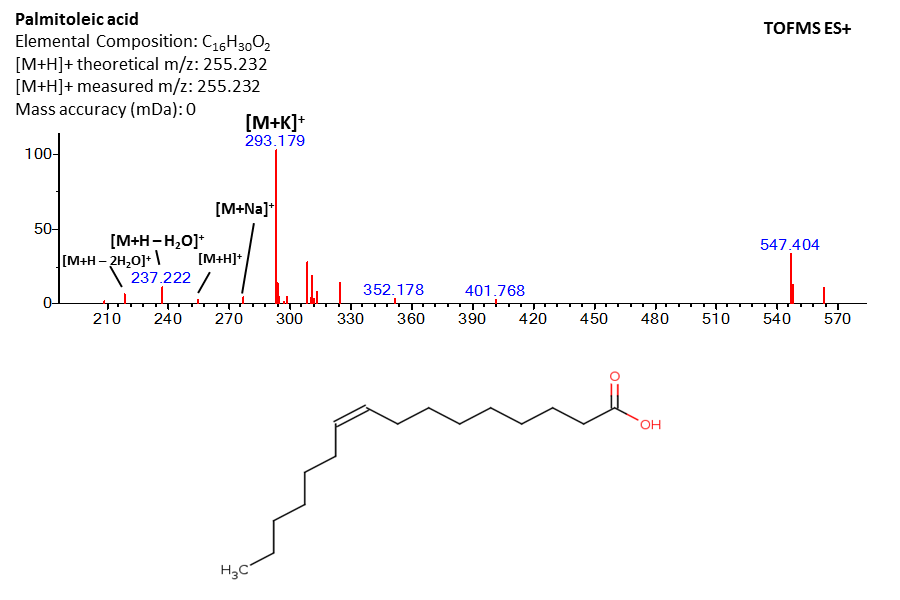


**Panel 7**: Metabolite eluting at 9.66 min represented by [M + H]^+^ = 431.388 tentatively identified as gamma-tocopherol.


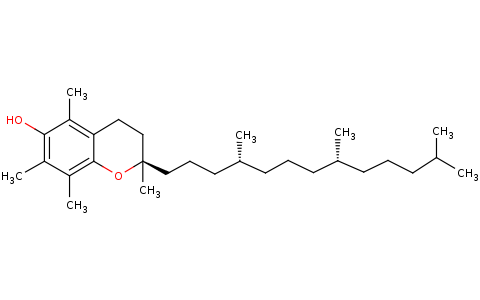


**Gamma-Tocopherol**

Elemental Composition: C_28_H_48_O_2_ [M+H]+ theoretical m/z: 431.388 [M+H]+ measured m/z: 431.388

Mass accuracy (mDa): 0

**TOFMS ES+**

**[M+H]^+^**

**Panel 8**: Metabolite eluting at 2.39 min represented by [M + H]^+^ = 585.271 tentativeley identified as bilirubin.

**Bilirubin**

Elemental Composition: C_33_H_36_N_4_O_6_ [M+H]+ theoretical m/z: 585.271 [M+H]+ measured m/z: 585.271

Mass accuracy (mDa): 0

**TOFMS ES+**

**[M+H]^+^**


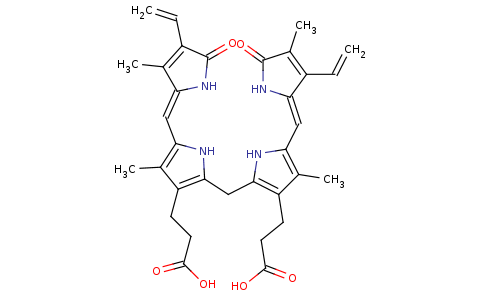


**299**

**Panel 9:** Metabolite eluting at 5.42 min represented by [M + Na]^+^ = 325.235 identified as monoacylglycerol (14:0).

**Panel 10:** Metabolite eluting at 7.20 min represented by [M + Na]^+^ = 379.282 identified as monoacylglycerol (18:1).

**Panel 11:** Metabolite eluting at 6.62 min represented by [M + H - H2O]^+^ = 337.273 identified as monoacylglycerol(18:2).
